# The “habitat-phytochemistry-pharmacological effect” nexus: a multidimensional review of ethnomedicinal *Salvia* in China

**DOI:** 10.3389/fphar.2026.1717261

**Published:** 2026-04-02

**Authors:** Linlin Zhao, Xing Yang, Sijing Su, Jihang Xie, Qian Zhang, Yusen Hou, Jiaxin Li, Xianzhe Li, Mingkun Meng, Ting Wang, Tingting Yan, Tong Xu, Yi Zhang

**Affiliations:** 1 School of Ethnic Medicine, Chengdu University of Taditional Chinese Medicine, Chengdu, Sichuan, China; 2 State Key Laboratory of Traditional Chinese Medicine Resources in Southwest China, School of Pharmacy, Chengdu University of Traditional Chinese Medicine, Chengdu, Sichuan, China; 3 Periplaneta Americana and Innovative Drugs Key Laboratory of Sichuan Province, Chengdu, China

**Keywords:** China, ethnomedicine, habitat-phytochemistry-pharmacological effect, multidimensional, *Salvia*

## Abstract

**Background:**

The genus *Salvia L.* constitutes a core medicinal resource in China’s diverse ethnic medical systems. However, a systematic and comparative understanding of how ecological adaptation-particularly among high-altitude ethnomedicinal species-translates into specific ethnomedicinal value remains lacking.

**Objective:**

To address this gap, we developed and validated a “habitat-phytochemistry-pharmacological effect” linkage model, testing the hypothesis that environmental stressors driven by altitudinal gradients induce chemical differentiation among *Salvia* species, which in turn underlies their distinct pharmacological properties and traditional therapeutic uses.

**Methods:**

We conducted a multidimensional analysis of 32 *Salvia* species documented in *the Dictionary of Chinese Ethnic Medicine*. This integrated systematic literature review, verification of altitudinal distributions using the Global Biodiversity Information Facility (GBIF), phenological characterization based on the Flora of China, and phytochemical profiling cross-referenced with the Human Metabolome Database (HMDB) and PubChem.

**Results:**

These species are used by 17 ethnic minority groups in China, primarily for cardiovascular and cerebrovascular diseases, gynecological disorders, and wound healing, guided by the principles of “activating blood circulation to resolve stasis” and “clearing heat and detoxifying.” Our findings support the habitat-adaptation hypothesis: biologically, species diverge into spring-flowering types (low-altitude, used predominantly by Miao and Zhuang communities) and summer-flowering types (high-altitude; widely employed in Tibetan medicine). Chemically, this divergence corresponds to marked compositional differences-low-altitude species are enriched in flavonoids, whereas high-altitude species accumulate higher levels of phenolic acids, consistent with adaptive responses to intense UV radiation at elevation. Functional compartmentalization was also observed: roots preferentially accumulate lipophilic diterpenoid quinones (associated with antitumor and antiplatelet effects) and hydrophilic phenolic acids (linked to antioxidant and antifibrotic activities), while aerial parts are rich in flavonoids (antibacterial and antitussive) and triterpenoids (immunomodulatory). Pharmacologically, *Salvia* species exhibit broad bioactivities - including anticancer, anti - inflammatory, hepatoprotective, and cardioprotective effects - mediated by multiple compound classes (terpenoids, phenolic acids, polysaccharides) acting through diverse pathways. Clinical evidence further corroborates a direct alignment between traditional efficacy concepts and molecular mechanisms: “activating blood circulation to resolve stasis” corresponds to diterpenoid quinone–mediated antitumor activity, and “clearing heat and detoxifying” aligns with phenolic acid-driven anti-inflammatory effects.

**Conclusion:**

This study successfully validates the “habitat-phytochemistry-pharmacological effect” linkage model, demonstrating a strong correlation between the ethnomedicinal value of *Salvia* species and their ecological traits, phytochemical profiles, and pharmacological actions. The model provides a robust framework for ethnopharmacology-guided natural product discovery. Future work should prioritize mechanistic studies of key active constituents and rigorous pharmacological validation of their traditional uses.

## Graphical Abstract

**Figure:**
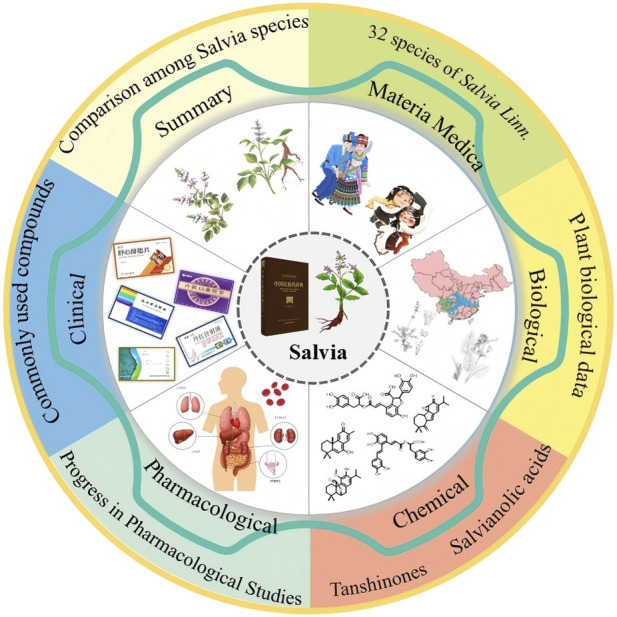

## 1 Introduction

The genus *Salvia* L. (Lamiaceae)-a globally distributed group comprising over 1,000 species-holds a central position in ethnopharmacological and phytotherapeutic research (Hu et al., 2023). China represents a biodiversity hotspot for East Asian *Salvia* species, harboring more than 80% of the regional diversity and exhibiting high levels of endemism (Hu et al., 2018). Beyond their ornamental value, these plants play an irreplaceable role in China’s ethnomedical systems (Li et al., 2011; Li C. Y. et al., 2022; Zhao C. et al., 2024).

This review focuses on the 32 medicinal *Salvia* species documented in the Dictionary of Chinese Ethnomedicines. We conducted a systematic literature search (2000–2025) using Web of Science, PubMed, and CNKI. Included studies met the following criteria: (1) focused on one of the 32 target species; (2) reported empirical data on botanical, chemical, or pharmacological properties. Botanical data-including altitudinal distributions-were further verified using the Global Biodiversity Information Facility (GBIF), and phenological traits were standardized according to the Flora of China. All chemical and pharmacological data were extracted exclusively from peer-reviewed empirical studies.

Our analysis centers on Salvia species used by 17 ethnic groups-including Tibetan, Miao, and Tujia communities-for treating cardiovascular and cerebrovascular diseases, gynecological disorders, trauma repair, and related conditions.

A defining feature of ethnomedicine is its foundational principle of “harmony between humanity and nature” (Zhao et al., 2012)-the notion that habitat characteristics (e.g., altitude, light, soil) directly influence the synthesis and accumulation of plant secondary Ingredientss, which in turn determine therapeutic efficacy. This has given rise to a distinct theoretical and practical framework for botanical drugal use (Zhao F. Y. et al., 2024). The “habitat-phytochemistry-pharmacological effect” (hereafter abbreviated as HPPE) relationship thus constitutes a key scientific basis for the pharmacological actions of these plants (Loussouarn et al., 2017). Altitudinal gradients and phenological traits significantly shape Ingredients distribution (Zhang et al., 2019), with high-altitude ultraviolet (UV) radiation acting as a critical environmental cue that induces phenolic acid biosynthesis-offering a strategy to optimize cultivation conditions for enhanced medicinal quality (Xing et al., 2024; Xu L et al., 2022).

Despite this potential, research on ethnomedicinal Salvia faces several challenges. Low bioavailability of active constituents remains a major bottleneck (Bi et al., 2016). Current studies have predominantly focused on low-altitude species such as Salvia miltiorrhiza, while the anti-hypoxic adaptive Ingredientss and immunomodulatory potential of high-altitude specialists-such as Salvia przewalskii-remain underexplored (Jang et al., 2017; Wang et al., 2020).

Integrating multidisciplinary approaches-including textual analysis, phytochemistry, and systems pharmacology-to elucidate the internal linkage among “ethnomedical application-biological traits-chemical constituents-pharmacological mechanisms-clinical efficacy” can not only provide mechanistic explanations for traditional concepts such as “activating blood circulation to resolve stasis” and “clearing heat and detoxifying,” but also establish an evidence-based foundation for resource development, quality control, and novel drug discovery. Such integration is essential to advance ethnomedicine from empirical practice toward precision and modernization.

Accordingly, this review addresses three key questions:

1. How do altitudinal gradients shape the chemodiversity of medicinal Salvia species?
2. To what extent can traditional ethnomedicinal uses be explained by habitat-specific chemical profiles?
3. What pharmacological mechanisms link specific constituents to recorded ethnomedicinal efficacies?

We hypothesize that the HPPE linkage in *Salvia* is causative rather than merely correlative: environmental pressures-particularly altitude-driven UV exposure-selectively activate distinct biosynthetic pathways, resulting in chemically divergent species that have been empirically selected by different ethnic groups to address health challenges prevalent in their local environments. This review systematically evaluates evidence from botany, phytochemistry, pharmacology, and clinical studies to test this hypothesis and proposes a predictive framework to guide future research and the sustainable utilization of *Salvia* resources.

## 2 Textual research on materia medica and ethnomedicinal history

### 2.1 Literatures records of *Salvia* plants

All 32 species are documented in the *Dictionary of Chinese Ethnomedicines*, providing a robust textual foundation for our analysis (Table 1). Among the 32 commonly used ethnomedicinal *Salvia* species, the primary medicinal parts are roots or whole botanical drugs. In terms of usage frequency, Tibetan communities utilize the greatest number of species (19), followed by the Miao and Tujia ethnic groups (5 species each), indicating that ethnic populations in Southwest China rely most extensively on *Salvia* for traditional healthcare. Representative root-based species include *Salvia miltiorrhiza* and *Salvia przewalskii*, whereas whole-botanical drug preparations are exemplified by *Salvia plebeia* and *Salvia chinensis*. These are predominantly employed to treat cardiovascular and cerebrovascular disorders (e.g., angina pectoris), gynecological conditions (e.g., irregular menstruation), and traumatic injuries (Li Y. et al., 2018). This usage pattern reflects core principles of traditional medicine-namely, “treating form with form” and “activating blood circulation to dredge collaterals”-highlighting its holistic therapeutic philosophy.

**TABLE 1:** Record of medicinal *Salvia* in dictionary of Chinese ethnomedicines.

No.	Latin name	Subgenus	Ethnic groups used	Medicinal part	Therapeutic effects
1	*Salvia bifidocalyx* C. Y. Wu & Y. C. Huang	Subg.*Salvia* Benth.	Tibetan	Root, flowers	Meridian stasis, palpitations, trauma, oral diseases, menstrual disorders, cough, Hepatitis
2	*Salvia bowleyana* Dumu	Subg.*Sclarea* Benth	She	Root	Treatment of irregular menstruation, amenorrhea, pelvic inflammation
3	*Salvia brachyloma* Stib.	Subg.*Salvia* Benth.	Tibetan	Root, flowers	Meridian stasis, palpitations, trauma, oral diseases, menstrual disorders, cough, Hepatitis
4	*Salvia brecilabra* Franch.	Subg.*Salvia* Benth.	Tibetan	Whole botanical drug	Eye diseases, corneal opacity
5	*Salvia campanulata* Wall. ex Benth	Subg.*Salvia* Benth.	Tibetan	Whole botanical drug	Eye diseases, corneal opacity
6	*Salvia castanea* diels.	Subg.*Salvia* Benth.	Tibetan	Whole botanical drug	Eye diseases, corneal opacity
7	*Salvia cavaleriei* H. Lévl.	Subg.*Sclarea* Benth	Dong	Whole botanical drug	Anti-inflammatory, analgesic, detumescent
​	​	​	Tujia	Whole botanical drug	Treatment of hematemesis, hemoptysis, epistaxis, bloody dysentery, metrorrhagia, traumatic bleeding
8	*Salvia cavaleriei* var. erythrophylla (Hemsl.)Stib.	Subg.*Salvia* Benth.	Miao	Whole botanical drug	Pulmonary tuberculosis with hemoptysis, dysentery, traumatic injury
​	​	​	Tujia	Whole botanical drug	Lung-heat cough, hemoptysis, traumatic injury, dysentery, epistaxis, metrorrhagia, hematochezia, traumatic bleeding, lung abscess, menstrual disorders
9	*Salvia cavaleriei* var. *simplicifolia* E.Stib.	Subg.*Salvia* Benth.	Tujia	Whole botanical drug	Lung-heat cough, hemoptysis, traumatic injury, dysentery, epistaxis, metrorrhagia, hematochezia, traumatic bleeding, lung abscess, menstrual disorders
​	​	​	Yao	Whole botanical drug	Cough with hemoptysis, metrorrhagia, bloody dysentery, wound bleeding, bronchitis
10	*Salvia chinensis* Benth.	Subg. *Allagospadonopsis* Briq	Qiang	Whole botanical drug	Pneumonia with hemoptysis, sore throat
​	​	​	Yao	Whole botanical drug	Dysphagia, cough and asthma, hepatitis, leukorrhagia, carbuncles, scrofula
​	​	​	Zhuang	Whole botanical drug	Cancer, heart/stomach pain, carbuncles
11	*Salvia deserta* Schang.	Subg.*Sclarea* Benth	Kazakh	Whole botanical drug	Epistaxis, gingival bleeding
12	*Salvia digitaloides* Diels	Subg.*Salvia* Benth.	Naxi	Root	Fever with thirst, sore throat, lung-heat cough, febrile rash, menstrual disorders, liver cirrhosis, heart pain, metrorrhagia, amenorrhea, abdominal mass, hepatosplenomegaly, restlessness, palpitations with insomnia, carbuncles, traumatic injury, joint pain, hernia, lumbago
13	*Salvia flava Forrest* ex Diels	Subg.*Salvia* Benth.	Tibetan	Root	Menstrual disorders, amenorrhea, angina, dysmenorrhea, metrorrhagia, joint pain, palpitations with insomnia, cardiovascular diseases, coronary heart disease, traumatic injury, blood stasis swelling, hepatitis, toothache
14	*Salvia glutinosa* L.	Subg.*Sclarea* Benth.	Tibetan	Root	Gastrorrhagia, pulmonary tuberculosis with hemoptysis, lung diseases, ulmonary tuberculosis with hemoptysis
15	*Salvia kiaometiensis* Levl.	Subg.*Salvia* Benth.	Tibetan	Root	Gastrorrhagia, pulmonary tuberculosis with hemoptysis, tendon and vein regeneration, heat stroke, intestinal heat, traumatic pain, meridian stasis, wound healing, menstrual disorders
16	*Salvia lankongensis* C.Y,Wu	Subg.*Salvia* Benth.	Bai	Root	Angina, myocardial sclerosis, dysmenorrhea, coronary heart disease, palpitations with insomnia, blood stasis, abdominal pain, insomnia
17	*Salvia mekongensi* Stib.	Subg.*Salvia* Benth.	Tibetan	Root, flower seeds	*Staphylococcus aureus*, diseases, corneal opacity, Eye diseases, corneal opacity
18	*Salvia miltiorrhiza* Bge.	Subg.*Sclarea* Benth	Achang	Root	Menstrual disorders, palpitations, joint pain
​	​	​	Buyi	Root	Liver cirrhosis
​	​	​	Jingpo	Root	Menstrual disorders, palpitations, joint pain
​	​	​	Mao Nan	Root:	Postpartum blood stasis abdominal pain
​	​	​	Meng	Root	Menstrual disorders, dysmenorrhea, postpartum blood stasis, ectopic pregnancy, hepatosplenomegaly, angina, restlessness with insomnia, carbuncles, blood heat, meridian heat, menstrual disorders, stomach “Boru” disease, intestinal heat diarrhea
​	​	​	Miao	Root	Menstrual disorders, blood stasis amenorrhea, stabbing pain due to blood stasis
​	​	​	Tujia	Root	Menstrual disorders, blood stasis amenorrhea, stabbing pain due to blood stasis, carbuncles, restlessness with insomnia, coronary heart disease, angina
​	​	​	Yao	Root and stem	Angina, palpitations with insomnia, menstrual disorders, dysmenorrhea, abdominal mass, blood stasis abdominal pain, joint pain, malignant carbuncles
​	​	​	Yi	Root	Palpitations with insomnia, menstrual disorders, asthma, menorrhagia
​	​	​	Tibetan	Root and rhizome	Gastrorrhagia, lung diseases, pulmonary tuberculosis with hemoptysis
19	*Salvia plebeia* R. Br.	Subg.*Sclarea* Benth	Lisu	Whole botanical drug	Hemoptysis, hematemesis, hematuria, metrorrhagia, ascites, leukorrhea, sore throat, carbuncles, hemorrhoids
​	​	​	Miao	Aerial parts	Common cold cough, vaginitis, mastitis
​	​	​	Naxi	Stem, leaves and flower spikes	Persistent high fever, cold, influenza, unexpressed measles, skin itching, hematemesis, epistaxis, scabies, headache, sore throat, urticaria, bronchitis, nephritic edema, carbuncles, mastitis, hemorrhoid pain, bleeding, traumatic injury, traumatic bleeding, snake bites
​	​	​	Tujia	Whole botanical drug	Common cold cough, sore throat, bronchitis, pulmonary tuberculosis with hemoptysis, abdominal edema, nephritic edema, hematochezia, thrombocytopenic purpura, hemorrhoid pain, carbuncles, dysentery; external use for trauma, contusions
​	​	​	Yao	Whole botanical drug	Sore throat, carbuncles; external use for mastitis, hemorrhoid pain
​	​	​	Zhuang	Whole botanical drug	Throat pain, bronchitis, edema, carbuncles; external use for mastitis, hemorrhoids
20	*Salvia pogonochila* diels	Subg.*Salvia* Benth.	Tibetan	Root	Gastrorrhagia, pulmonary tuberculosis with hemoptysis, tendon and vein regeneration, heat stroke, intestinal heat, traumatic pain, meridian stasis, wound healing, menstrual disorders
21	*Salvia prattii* Hemsl.	Subg. *Salvia* Benth	Tibetan	Root and whole botanical drug	Oral diseases, liver heat, toothache, warm-type dental diseases, Gastrorrhagia, pulmonary tuberculosis with hemoptysis, tendon and vein regeneration, heat stroke, intestinal heat, traumatic pain, meridian stasis, wound healing, menstrual disorders
22	*Salvia prionitis* Hance	Subg.*Sclarea* Benth	Zhuang	Whole botanical drug	Pharyngitis, cough, dysentery
23	*Salvia przewalskii* Maxim.	Subg.*Salvia* Benth.	Naxi	Root	Burns, pain relief and muscle regeneration, heart pain, metrorrhagia, amenorrhea, restlessness, palpitations with insomnia, carbuncles, traumatic injury, joint pain, hernia, lumbago
​	​	​	Yi	Root	Moistening lung and quenching thirst
​	​	​	Tibetan	Flowers	Chronic cough, hepatitis, Gastrorrhagia, pulmonary tuberculosis with hemoptysis, hepatitis, pneumonia, cough, lung heat; Gastrorrhagia, pulmonary tuberculosis with hemoptysis, lung diseases, chest pain due to restlessness, dizziness due to blood deficiency, liver diseases, oral ulcers, tendon and vein regeneration, heat stroke, intestinal heat, traumatic pain, wound healing; inflorescence: Hepatitis, lung heat, cough, liver diseases, oral ulcers, toothache
24	*Salvia przewalskii* var.mandarinorum (Diels) E.Peter.	Subg.*Salvia* Benth.	Yi	Root	Removing blood stasis and relieving pain, activating blood circulation and regulating menstruation, clearing heart and removing vexation
​	​	​	Tibetan	Flowers	Chronic cough, hepatitis; root and inflorescence: Gastrorrhagia, lung diseases, pulmonary tuberculosis with hemoptysis, hepatitis, cough
25	*Salvia pseudopallida* Epling	Subg.*Jungia (Moench)* Briq	Tibetan	Whole botanical drug	Pallor of face and gums due to anemia, physical weakness, restoring balance of three humors
26	*Salvia roborowskii* Maxim.	Subg.*Salvia* Benth.	Tibetan	Whole botanical drug	Hepatitis, wind-fire toothache, pneumonia, pulmonary tuberculosis, hemoptysis, Eye diseases, parasitic diseases
27	*Salvia scapiformis* Hance	Subg. *Allagospadonopsis* Briq	Yao	Whole botanical drug	Blood deficiency amenorrhea, menstrual disorders, leukorrhea, blood deficiency migraine, overstrain body pain, weakness and emaciation
28	*Salvia smithii* Stib.	Subg.*Salvia* Benth.	Tibetan	Root	Hepatitis, toothache
29	*Salvia splendens* Ker.-Gawl.	Subg.*Jungia (Moench)* Briq	Miao	Whole botanical drug	Physical weakness, traumatic injury, carbuncles
30	*Salvia trijuga* Diels	Subg.*Sclarea* Benth.	Naxi	Root	Menstrual disorders, dysmenorrhea, blood deficiency amenorrhea, kidney deficiency lumbago, impotence, metrorrhagia, neurasthenia with insomnia and restlessness, early liver cirrhosis
​	​	​	Tibetan	Root	Menstrual disorders, dysmenorrhea, blood deficiency amenorrhea, kidney deficiency lumbago, neurasthenia, insomnia, impotence, metrorrhagia, meridian stasis, palpitations, trauma, oral diseases, menstrual disorders, amenorrhea, blood stasis abdominal pain, abdominal mass; cough, hepatitis
31	*Salvia wardii* Pet-stib	Subg.*Salvia* Benth.	Tibetan	Flowers, leaves, stems, fruits, whole botanical drug	Eye diseases, parasitic diseases
32	*Salvia yunnanensis* C,H. Wright	Subg.*Sclarea* Benth.	Miao	Root	Menstrual disorders, dysmenorrhea, amenorrhea, lochial abdominal pain, blood deficiency limb numbness, insomnia, amnesia, palpitations, abdominal mass, chest bi colic, restlessness with internal heat, joint pain, hernia, carbuncles, erysipelas, metrorrhagia, hematemesis, epistaxis, hemoptysis, palpitations, mastitis, sores, traumatic swelling, bronchitis
​	​	​	Yi	Root	Menstrual disorders, metrorrhagia, abdominal mass, amenorrhea, mastitis, postpartum high fever, abdominal mass, blood stasis swelling, amenorrheal abdominal pain, carbuncles, traumatic bleeding, epistaxis, contusions, abdominal mass, dizziness and neurasthenia, persistent chronic hepatitis, thromboangiitis obliterans, hepatosplenomegaly in advanced schistosomiasis, coronary heart disease

Analysis of the *Dictionary of Chinese Ethnomedicines* reveals that Southwest China-encompassing Yunnan, Guizhou, Sichuan, and Tibet-serves as the core region for both the distribution and ethnomedicinal application of *Salvia* species, reflecting the traditional “local materia medica” principle. Among ethnic groups, Tibetans are the predominant users (19 species, ∼60%), primarily employing high-altitude taxa such as *Salvia przewalskii* and *Salvia prattii*. Their applications align closely with health challenges prevalent in plateau environments, including trauma repair, lung-heat cough, and hepatosplenomegaly. In contrast, the Miao and Tujia groups (5 species each) mainly utilize low-elevation, whole-botanical drug species like *Salvia cavaleriei* and *Salvia plebeia* to address traumatic injuries, gynecological inflammation, and respiratory infections-demonstrating habitat-adaptive medicinal practices.

Regarding medicinal parts, roots (∼60% of uses) are primarily associated with “activating blood circulation and resolving stasis,” targeting cardiovascular and cerebrovascular diseases, gynecological blood stasis syndromes, and internal injuries. Whole botanical drugs (∼40%) emphasize “clearing heat and detoxifying,” and are mainly applied to external pathogenic inflammations, superficial infections, and traumatic bleeding. This therapeutic logic-“roots for internal ailments, whole botanical drugs for external conditions”-not only embodies the traditional concept of “treating form with form” but also underscores the intrinsic link between ecological adaptation and medicinal function.

### 2.2 Application of regionally specific *Salvia* medicinal materials

The medicinal use of *Salvia* in China dates back to the Qin Dynasty. Ancient pharmacopeias-including *Shennong’s Materia Medica*, *Compendium of Materia Medica*, and regional floras-document 25 *Salvia* species as medicinally relevant (Wang Y. et al., 2023). Among them, only *Salvia miltiorrhiza* is officially included in the *Chinese Pharmacopoeia* (2025 edition), where it is described as capable of “removing blood stasis, generating new blood, activating blood circulation, and regulating menstruation.” Its therapeutic history traces to the Eastern Han Dynasty’s *Shennong’s Materia Medica*, where it was classified as a “superior-grade” botanical drug. Today, it remains a cornerstone in clinical TCM treatment of cardiovascular and gynecological disorders (Ren et al., 2019).

In ethnopharmacological practice, *Salvia przewalskii*, *Salvia yunnanensis*, and *Salvia bowleyana* are frequently used as regional substitutes for *Salvia miltiorrhiza* in Southwest, Northwest, and South China, respectively (Xu et al., 2018; Zhumaliyeva et al., 2023). Beyond this flagship species, multi-ethnic *Salvia* materials constitute vital constituent of local medical systems (Tao et al., 2019). For instance, *Salvia przewalskii*, recorded in the *Jingzhu Materia Medica*, is native to Sichuan and Tibet (Weng et al., 2015). It is used to clear liver heat, treat oral diseases, and concurrently regulate menstruation, activate blood circulation, resolve stasis, and relieve pain-often combined with other botanical drugs to address disorders stemming from impaired qi and blood flow (Li et al., 2010).

*Salvia prionitis* is a characteristic Zhuang medicinal plant, commonly prescribed for colds, fevers, and sore throats due to its wind-dispersing and heat-clearing properties. *Salvia yunnanensis*, utilized by Yi and Bai communities in Yunnan (Guan et al., 2020), exhibits heat-clearing, detoxifying, blood-activating, stasis-resolving, anti-inflammatory, and analgesic effects, showing marked efficacy in treating traumatic injuries and swelling-pain syndromes.

*Salvia bowleyana* is a key She ethnic remedy for rheumatic pain, numbness, and joint dysfunction. Meanwhile, *Salvia deserta*, endemic to Xinjiang, is used as a whole botanical drug in Kazakh and Uyghur folk medicine for its ability to clear heat, detoxify, relieve cough, resolve phlegm, reduce swelling, and promote diuresis (Chen et al., 2020; Zhang X. Y. et al., 2025; Li Z. M. et al., 2018).

**FIGURE 1:**
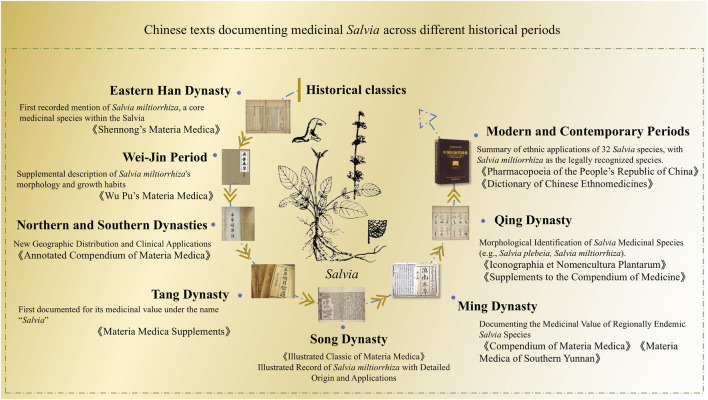
Timeline of medicinal *Salvia* documentation in classical Chinese ethnic medical texts.

Figure 1 summarizes the documentation of medicinal *Salvia* species in classical Chinese ethnic medical texts. This diagram, compiled based on sources such as *Shennong’s Materia Medica*, illustrates the historical documentation of medicinal *Salvia* in ancient texts. Notably, *Salvia miltiorrhiza* remains the sole representative of the genus formally recognized in the *Pharmacopoeia of the People’s Republic of China*, underscoring a gap between official pharmacopeial standards and rich ethnomedicinal diversity. These historical and ethnobotanical records provide critical context for interpreting traditional applications and guiding future evidence-based validation.

## 3 Biological characteristics and ecological adaptability research

If altitude constitutes a key driver of chemical differentiation, then *Salvia* species distributed across distinct elevational gradients should exhibit discernible morphological and phenological adaptive traits. This section aims to verify whether such biological differentiation aligns with the hypothesized patterns of chemical divergence and associated medicinal trait variation.

Most Chinese medicinal *Salvia* species are perennial erect botanical drugs, ranging from 30 to 80 cm in height, with fleshy, thickened roots covered by bright red or purplish-brown epidermis. Their stems are quadrangular and glandular-hairy; the leaves are opposite and odd-pinnately compound, bearing ovate to lanceolate leaflets with crenate margins that are pubescent on both sides. Verticillasters are arranged in terminal racemes, and the corollas are purple-blue and bilabiate, whereas the nutlets are elliptic. These plants flower from May to August and fruit from August to September (Liu et al., 2023; Chen et al., 2018).

*Salvia* plants have long been documented in classical medical texts. Modern research further confirms that the material basis of their pharmacological efficacy is highly consistent with their ecological adaptability, with altitude playing a pivotal role in shaping their biological characteristics (Li Y. et al., 2018; Liu et al., 2023).

Among these adaptations, altitude-driven phenological differentiation is particularly pronounced. Low-altitude species (120–1,500 m), such as *Salvia miltiorrhiza* and *Salvia cavaleriei*, form small-flowered groups that bloom in spring (April–June), adapting to the warmer temperatures and shorter photoperiods typical of lowland environments. In contrast, high-altitude species (2,100–4,500 m), including *Salvia przewalskii* and *Salvia prattii*, produce large-flowered inflorescences that bloom in summer (July–August), synchronizing their reproductive phase with the extended growing season and intense solar radiation characteristic of alpine zones. This phenological divergence reflects distinct strategies for accumulated temperature utilization and photoperiodic regulation across elevational gradients (Chen et al., 2018; Geng et al., 2020).

Floral color and morphology also exhibit altitude-related specialization. High-altitude medicinal *Salvia* species-such as *Salvia prattii*-typically display dark purple or vivid yellow corollas, while *Salvia yunnanensis* and *Salvia wardii* feature blue flowers (Duan et al., 2019a). These traits are thought to enhance pollinator attraction in high-elevation ecosystems dominated by bumblebees. Conversely, low-altitude species primarily rely on butterfly pollination, and their floral colors and shapes are optimized accordingly (Moein et al., 2023; Sazatornil et al., 2023). Notably, pink and white flower morphs are rare among medicinal *Salvia*.

Taking *Salvia przewalskii* as an example, significant differences in photosynthetic capacity have been observed along altitudinal gradients in the eastern Qinghai-Tibet Plateau. This species modulates its photosynthetic machinery to cope with high ultraviolet (UV) radiation and pronounced diurnal temperature fluctuations, exhibiting a plastic response to high-altitude stress (Xing et al., 2024). Such physiological adaptability directly influences biomass accumulation and secondary Ingredients biosynthesis. Specifically, high-altitude species accumulate elevated levels of phenolic acids (e.g., salvianolic acids) as a defense against UV-induced oxidative stress, whereas low-altitude species like *Salvia miltiorrhiza* preferentially synthesize flavonoids (Xu L et al., 2022; Yang et al., 2012; Yin et al., 2020).

Meteorological factors associated with elevation-such as temperature, precipitation, and UV intensity-directly regulate both biomass production and active constituent content in *Salvia miltiorrhiza* across different climatic zones (Zhang et al., 2016a). High-altitude habitats, in particular, appear to favor the synthesis of stress-responsive phytochemicals.

From a genetic and evolutionary perspective, the chemical diversity of abietane-type diterpenoids within the genus *Salvia* is partially attributed to functional divergence and neofunctionalization of key biosynthetic enzyme genes, such as *CYP76AK*. We hypothesize that this genetic-level differentiation may stem from long-term adaptive evolution to distinct altitudinal niches-particularly in response to selective pressures imposed by UV radiation intensity and thermal variability. For instance, the intense oxidative stress prevalent in high-altitude environments may have driven the selection of diterpenoids with specific structural modifications, thereby indirectly shaping the evolutionary trajectory of their biosynthetic pathways. However, this hypothesis requires direct empirical validation through comparative genomics and population genetic analyses of closely related species distributed across elevational gradients (Wu et al., 2021; Huang et al., 2025).

Such genetic variation underpins the observed differences in chemical profiles among species and enables niche specialization-for example, allowing *Salvia castanea* to thrive in specific mid-to-high elevation habitats (Wang et al., 2012; Liao et al., 2022; Chen et al., 2022). Functional studies further support this link: overexpression of the *ScWRKY35* gene from *Salvia castanea* in *Salvia miltiorrhiza* enhances stress tolerance and modulates Ingredients production, highlighting the genetic mechanisms that facilitate adaptation to diverse altitudinal environments (Zhang et al., 2024; Zhang et al., 2016b).

Despite these insights, the cultivation of high-altitude *Salvia* species remains challenging due to their dependence on specific environmental conditions-including UV radiation levels above 3,000 m and daily temperature fluctuations exceeding 15 °C. In contrast, 16 low-altitude species (e.g., *Salvia miltiorrhiza* and *Salvia bowleyana*) have been successfully domesticated, underscoring the critical role of altitude in determining cultivation feasibility (Table 2).

**TABLE 2:** Biological information of Chinese medicinal Salvia.

No.	Latin name	Morphological characteristics	Flowering season	Growth habitat	Geographic distribution
1	*Salvia.* C. Y. Wu and Y. C. Huang	Perennial botanical drug; height ∼33 cm; flowers blue or blue-purple	July	Rocky mountains, altitude 3,500 m	Northwestern Yunnan
2	*Salvia bowleyana* Dumu	Perennial botanical drug; height 1 m; corolla pale purple, purple to blue-purple	March-July	Mountains, valleys, roadsides, forests or water edges at 30–960 m altitude	Zhejiang, Hunan, Jiangxi, Fujian, Guangdong, Guangxi, *etc.*
3	*Salvia brachyloma* Stib.	Perennial botanical drug; height 20–57 cm; corolla pale purple	June-July	Under forests, forest-edge grasslands or meadows at 3,200–3,800 m	Northwestern Yunnan and southwestern Sichuan
4	*Salvia brecilabra* Franch.	Perennial botanical drug; height 60 cm; corolla blue-purple	July-August	Slopes, grasslands and forest lands at 3,200–3,850 m	Western Sichuan
5	*Salvia campanulata* Wall. ex Benth	Perennial botanical drug; height 43–80 cm; corolla yellow	July-September	Forest edges at ∼2,300 m	Northwestern Yunnan, China; also in Sikkim, Nepal, northern India
6	*Salvia castanea* diels.	Perennial botanical drug; height 30–65 cm; corolla yellow	May-September	Sparse forests, forest edges or forest-edge grasslands at 2,500–2,800 m	Northwestern Yunnan and southwestern Sichuan
7	*Salvia cavaleriei* Lévl.	Annual botanical drug; height 12–32 cm; corolla blue-purple or purple	July-September	Rocky slopes, forests, ditch edges at 530–1,300 m	Sichuan, Guizhou, Guangxi, Guangdong, China
8	*Salvia cavaleriei* var. erythrophylla (Hemsl.)Stib.	Annual botanical drug; height 12–32 cm; flowers dark purple or white, corolla blue-purple or purple	July-September	Forests, roadsides, grass slopes at 200–700 m	Hubei, Sichuan, Shaanxi, Hunan, Guangxi, Yunnan
9	*Salvia cavaleriei* var. simplicifolia Stib.	Perennial botanical drug; height 12–32 cm; flowers purple or magenta	July-September	Slopes, forests or ditch edges at 460–2,700 m	Hubei, Hunan, Jiangxi, Guangdong, Guangxi, Guizhou, Yunnan, Sichuan, Fujian, etc.
10	*Salvia chinensis* Benth.	Annual botanical drug; height 60 cm; flowers blue-purple or purple	August-January	Forested areas or grasslands on slopes or flatlands at 120–500 m	Shandong, southern Jiangsu, southern Anhui, Zhejiang, Hubei, Jiangxi, Hunan, Fujian, Taiwan, northern Guangdong, northeastern Guangxi, Sichuan, etc.
11	*Salvia deserta* Schang.	Perennial botanical drug; height ∼70 cm; corolla blue-purple to purple	June-January	Field wastelands, ditch edges, sandy grasslands and forests at 270–1850 m	Northern Xinjiang; also in Russia, Kazakhstan, Kyrgyzstan, etc.
12	*Salvia digitaloides* diels	Perennial botanical drug; height 30–60 cm; corolla yellow with pale purple spots	April-June	Under mountain pine forests and wasteland grasslands at 2,500–3,600 m	Yunnan, southwestern Sichuan
13	*Salvia flava* Forrest ex diels	Perennial botanical drug; height 20–50 cm; corolla yellow	July	Under forests and slope grasslands at 2,500–4,000 m	Northwestern Yunnan, southwestern Sichuan
14	*Salvia glutinosa* L.	Perennial botanical drug; height 1–1.25 m; flowers pale yellow	June-August	Deciduous and mixed forest areas at 100–1,600 m	Tibet; West Asia, Southern Europe, Afghanistan, Pakistan, India, Sikkim, Bhutan
15	*Salvia kiaometiensis* Levl.	Perennial botanical drug; height 25–50 cm; corolla purplish-brown or red	May-November	Slope grasslands at 2,500–3,200 m	Sichuan, Yunnan
16	*Salvia lankongensis* C.Y,Wu	Perennial botanical drug; height 23–28 cm; corolla blue	July	Grasslands and shrubs at 3,780 m	Northwestern Yunnan
17	*Salvia mekongensi* Stib.	Annual botanical drug; height 30 cm; corolla yellow	June-September	Slope grasslands at 2,800–4,100 m	Northwestern Yunnan
18	*Salvia miltiorrhiza* Bge.	Perennial botanical drug; height 30–100 cm; flowers purple or blue-purple	May-September	Slopes, forest grasslands or ditch edges at 120–1,300 m	Liaoning, Hebei, Shanxi, Shaanxi, Ningxia, Gansu, Shandong, Jiangsu, Anhui, Zhejiang, Fujian, Jiangxi, Henan, Hubei, Hunan, Sichuan, Guizhou, etc.
19	*Salvia plebeia* R. Br.	Perennial botanical drug; height 15–90 cm; corolla pale red, pale purple, purple, blue-purple to blue, rarely white	April-May	Wet soils on slopes, roadsides, ditch edges, fields	China, Korea, Japan, Afghanistan, India, Myanmar, Thailand, Vietnam, Malaysia, Australia, etc.
20	*Salvia pogonochila* diels	Perennial botanical drug; height 30–50 cm; corolla blue-purple	July-September	Alpine meadows	Sichuan
21	*Salvia prattii* Hemsl.	Perennial erect botanical drug; height ∼45 cm; corolla red or blue-purple	July-September	Slope grasslands at 3,750–4,800 m altitude	Western and northwestern Sichuan, southern Qinghai
22	*Salvia prionitis* Hance	Annual botanical drug; height 20–43 cm; corolla blue-purple	June-August	Slopes, sunny grasslands and roadsides at 100–800 m altitude	Guangxi, Jiangxi, Fujian, Guangdong, etc.
23	*Salvia przewalskii Maxim.*	Perennial botanical drug; height 60 cm; corolla magenta	May-September	Under forests, forest edges, ditch edges and grasslands at 2,100–3,500 m altitude	Southern to eastern Tibet, western Gansu, western Sichuan, northwestern Yunnan
24	*Salvia przewalskii* var.mandarinorum (diels) E.Peter.	Perennial botanical drug; height 60 cm; corolla magenta	July-September	Forest edges, under shrubs, ditch edges and roadsides at 2,100–3,500 m	Southern to eastern Tibet, western Gansu, western Sichuan, northwestern Yunnan
25	*Salvia pseudopallida* Epling	Perennial botanical drug; height 30–100 cm; flowers blue-purple, pale red or pale blue	June-September	Slopes, roadsides, shaded grasslands, water edges and forests at 220–1,100 m	Zhejiang, southern Anhui, Jiangsu, Jiangxi, Hubei, Fujian, Taiwan, Guangdong, Guangxi
26	*Salvia roborowskii* Maxim.	Perennial botanical drug; height 30–90 cm; flowers yellow	June-August	Slope grasslands, forest edges, ditch edges, field edges at 2,500–4,000 m	Qinghai, Sichuan, Gansu, Yunnan, Tibet
27	*Salvia scapiformis* Hance	Annual botanical drug; height 20–26 cm; flowers purple	April-May	Valley forests, mountains, roadsides, sparse forests or village edges at 300–1,250 m	Guangdong, Guangxi, Guizhou, Zhejiang, Fujian, Jiangxi, Hunan
28	*Salvia smithii* Stib.	Perennial botanical drug; height 6–40 cm; corolla colors include orange-yellow,deep blue, purple, *etc.*	April-June	Forests, shrubs, grasslands or slopes at 2,550–3,300 m	Southwestern Sichuan, northwestern and northeastern Yunnan, northwestern Guizhou
29	*Salvia splendens* Ker.-Gawl.	Perennial subshrubby botanical drug; height 20–90 cm; flowers red	July-October	Warm, humid climate with ample sunlight	Brazil; Beijing, Tianjin, Hebei, Shanxi, Inner Mongolia, Liaoning, Jilin, Heilongjiang, China
30	*Salvia trijuga* diels	Perennial botanical drug; height 30–60 cm; corolla blue-purple with yellow spots	July-September	Slopes, valleys, ditch edges, shrubs, forests or grasslands at 1900–3,900 m	Northwestern Yunnan, southwestern Sichuan, southeastern Tibet
31	*Salvia wardii* Pet-stib	Perennial botanical drug; height 40–75 cm; flowers blue with white lower lip or pale purple	May-August	Alpine gravel grasslands and shrubs at 3,600–4,500 m	Eastern Tibet, China
32	*Salvia yunnanensis* C,H. Wright	Perennial botanical drug; height 10–30 cm; corolla purple or magenta	June-August	Slope grasslands, forest edges or sparse forests at 1800–2,900 m	Yunnan, southwestern Sichuan, western Guizhou

**FIGURE 2:**
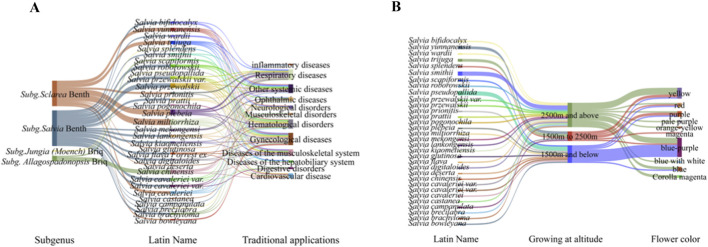
Sankey diagrams illustrating multidimensional correlations in Chinese medicinal *Salvia* species. **(A)** Flow diagram depicting associations among specific *Salvia* species, their documented traditional therapeutic effects (e.g., “activating blood circulation to resolve stasis,” “clearing heat and detoxifying”), and the corresponding modern pharmacological activity categories reported in the literature. **(B)** Flow diagram linking taxonomic classification (Subgenus), corolla color, ecological distribution (altitudinal zone), and cultivation status.

Figure 2 includes two Sankey diagrams: Sankey A: “Traditional Therapeutic Effects of Chinese Medicinal *Salvia* Plants,” illustrating the flow from specific species to documented traditional uses (e.g., “activating blood circulation to resolve stasis”) and corresponding modern pharmacological activity categories. Sankey B: A multidimensional flow diagram linking taxonomic classification (subgenus), corolla color, ecological distribution (altitudinal zone), and cultivation status.

**FIGURE 3:**
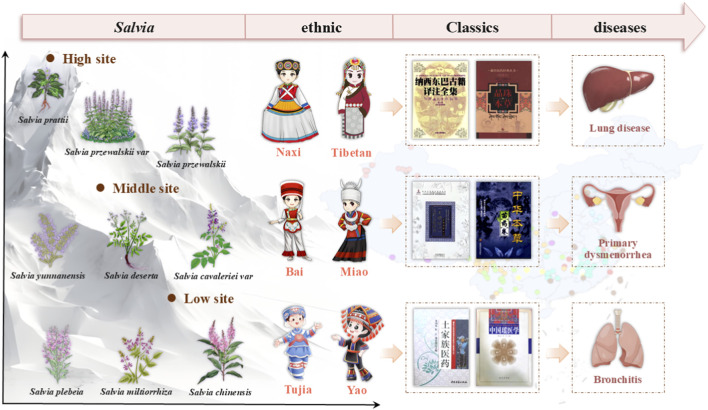
Relationship diagram of Chinese *Salvia* altitude-medicinal *Salvia* plant-use ethnicity-indicative diseases.

**FIGURE 4:**
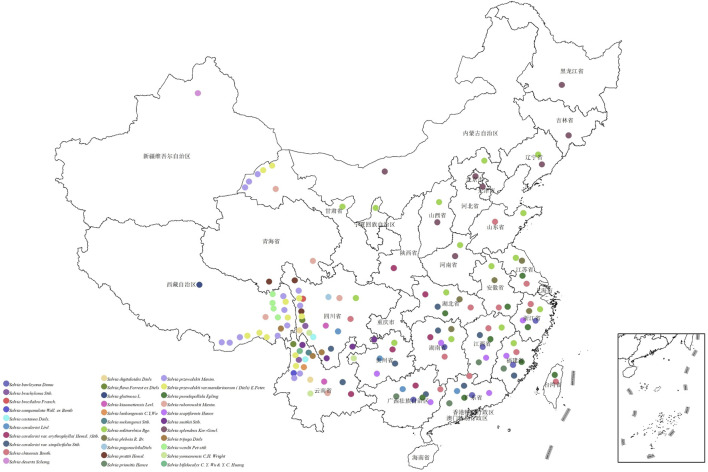
Geographical distribution Map of Chinese medicinal *Salvia*.

Figure 3 presents an integrative relationship diagram that logically connects four key dimensions: growth altitude of Chinese *Salvia* species, specific medicinal taxa, the ethnic groups utilizing them, and their associated indicative diseases. This visualization offers a comprehensive systems-level view of how ecological, botanical, cultural, and therapeutic factors are interwoven in traditional *Salvia*-based medicine Figure 4.

It should be noted that the primary data source for this study-the *Dictionary of Chinese Ethnic Medicine*-may underrepresent recently emerged or orally transmitted ethnomedical knowledge and may overrepresent ethnic groups with established written traditions (e.g., Tibetan and Miao). Additionally, ecological occurrence data (from GBIF and *Flora of China*) suffer from sampling biases, particularly in under-surveyed high-altitude and remote regions. Coarse-scale altitude classifications may also obscure critical microhabitat heterogeneity. Furthermore, chemical and phenological research coverage remains uneven across species. Collectively, these limitations suggest that observed HPPE correlations should be interpreted with caution. Future studies should integrate targeted field surveys with high-resolution environmental and metabolomic analyses to strengthen causal inference.

## 4 Chemical ingredientss

The chemical profiles of *Salvia* species are remarkably diverse and exhibit substantial variation across taxa and environmental contexts-particularly in relation to altitudinal gradients. These phytochemical differences underpin the distinct pharmacological properties and therapeutic applications associated with different *Salvia*-based remedies.

### 4.1 Characteristic chemical ingredientss

Characteristic secondary Ingredientss in medicinal *Salvia* species display strong species-specificity and environmental responsiveness. Four major classes dominate their phytochemistry: phenolic acids, flavonoids, terpenoids (including diterpenes and triterpenes), and volatile oils (Wang J. et al., 2019). However, the relative abundance and structural diversity of these compounds vary significantly depending on plant organ and elevation of origin.

**FIGURE 5:**
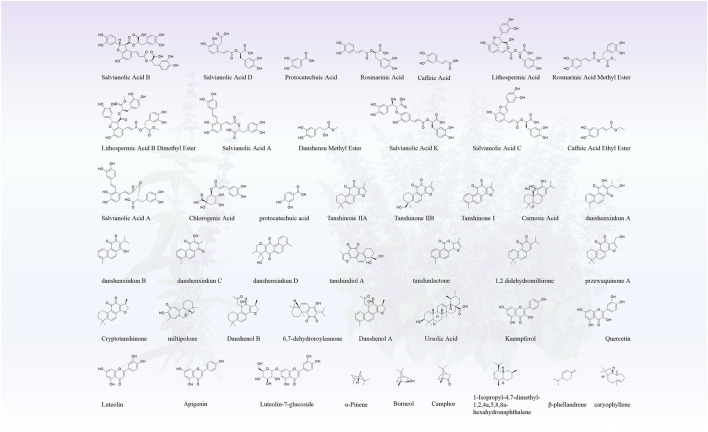
Representative chemical structures of major bioactive constituents found in Chinese medicinal *Salvia* species.

Roots-the primary medicinal tissue for many *Salvia* species-are enriched in lipophilic abietane-type diterpenoid quinones (e.g., tanshinones) and water-soluble phenolic acids (e.g., salvianolic acids), both of which contribute to antioxidant, anti-inflammatory, and antifibrotic activities. In contrast, aerial parts (leaves, stems, and flowers) predominantly accumulate flavonoids and triterpenoids, which are often linked to antimicrobial and immunomodulatory effects (Xia et al., 2023; Han et al., 2023; Wang F. et al., 2024). Comprehensive summaries of species-specific chemical constituents are provided in Tables 3, 4. Figure 5 presents a graphical abstract highlighting key bioactive molecules representative of medicinal *Salvia* species.

**TABLE 3:** Distribution of chemical Ingredients types in medicinal plants of the genus *Salva* in China.

No.	Latin name	Phenolic acids	Flavonoids	Terpenoids	Volatile oils	Others	References
1	*Salvia bifidocalyx* C. Y. Wu and Y. C. Huang	-	-	-	-	-	​
2	*Salvia bowleyana* Dumu	II	II	IV	-	I	Zhao et al. (2023), Chen (2017)
3	*Salvia brachyloma* Stib.	-	-	-	-	-	​
4	*Salvia brecilabra* Franch.	-	-	-	-	-	​
5	*Salvia campanulata* Wall. ex Benth	-	-	-	-	-	​
6	*Salvia castanea* Diels.	III	II	IV	-	I	Yang et al. (2023)
7	*Salvia cavaleriei* Lévl.	IV	III	II	II	II	Yang et al. (2023)
8	*Salvia cavaleriei* var. erythrophylla (Hemsl.)Stib.	-	-	-	-	-	​
9	*Salvia cavaleriei* var. simplicifolia Stib.	-	-	-	-	-	​
10	*Salvia chinensis* Benth.	II	III	II	II	II	Lei et al. (2010)
11	*Salvia deserta* Schang.	II	II	III	II	I	Hua (2016)
12	*Salvia digitaloides* Diels	-	-	-	-	-	​
13	*Salvia flava Forrest ex* Diels	-	-	-	-	-	-
14	*Salvia glutinosa* L.	I	-	-	-	-	Nicolescu et al. (2022)
15	*Salvia kiaometiensis* Levl.	-	-	-	-	-	​
16	*Salvia lankongensis* C.Y,Wu	-	-	-	-	-	​
17	*Salvia mekongensi* Stib.	-	-	-	-	-	​
18	*Salvia miltiorrhiza* Bge.	IV	II	VI	II	IV	Lan et al. (2025)
19	*Salvia plebeia* R. Br.	IV	II	VI	II	II	Chen et al., 2020;
20	*Salvia pogonochila* Diels	-	-	-	-	-	​
21	*Salvia prattii* Hemsl.	II	II	III	II	I	Bian (2023)
22	*Salvia prionitis* Hance	I	-	IV	I	I	Chen et al. (2021)
23	*Salvia przewalskii* Maxim.	IV	I	IV	III	II	Chen et al. (2022)
24	*Salvia przewalskii* var.mandarinorum (Diels) E.Peter.	-	-	-	-	​	​
25	*Salvia pseudopallida* Epling	-	-	-	-	-	​
26	*Salvia roborowskii* Maxim.	-	-	-	-	-	​
27	*Salvia scapiformis* Hance	-	-	-	-	-	​
28	*Salvia smithii* Stib.	-	-	-	-	-	​
29	*Salvia splendens Ker*.-Gawl.	III	-	III	II	II	Zhao C. et al. (2024)
30	*Salvia trijuga* Diels	-	-	-	-	-	​
31	*Salvia wardii* Pet-stib	-	-	-	-	-	​
32	*Salvia yunnanensis* C,H. Wright	III	III	IV	III	II	Wu (2012)

I indicates the number of compounds is 1–15; II indicates 16–30; III indicates 31–45; IV indicates 45–60; V indicates 61–75; VI indicates ≥75; -. Compounds not statistically listed in the table are derived from the collation results of retrieved literature.

**TABLE 4:** Characteristic Chemical Ingredientss of *Salvia* species.

No.	Chemical formula	Molecular formula	Category	Representative species	References
1	Salvianolic acid B	C_36_H_30_O_16_	Phenolic acids	*Salvia castanea* Diels., *Salvia miltiorrhiza* Bge., *Salvia przewalskii* Maxim., *Salvia cavaleriei* Lévl.	Xu M. et al. (2022), Lan et al. (2025)
2	Salvianolic acid D	C_20_H_18_O_10_	Phenolic acids	*Salvia miltiorrhiza* Bge., *Salvia przewalskii* Maxim.	Lan et al. (2025), Zhao C. et al. (2024)
3	Protocatechuic acid	C_7_H_6_O_4_	Phenolic acids	*Salvia miltiorrhiza* Bge., *Salvia przewalskii* Maxim.	Lan et al. (2025)
4	Rosmarinic acid	C_18_H_16_O_8_	Phenolic acids	*Salvia miltiorrhiza* Bge., *Salvia przewalskii* Maxim.	Zhao C. et al. (2024)
5	Caffeic acid	C_9_H_8_O_4_	Phenolic acids	*Salvia cavaleriei* Lévl., *Salvia deserta* Schang., *Salvia miltiorrhiza* Bge., *Salvia przewalskii* Maxim., *Salvia prattii* Hemsl.	Zhao C. et al. (2024)
6	Lithospermic acid	C_27_H_22_O_12_	Phenolic acids	*Salvia miltiorrhiza* Bge., *Salvia przewalskii* Maxim.	Zhao C. et al. (2024)
7	Rosmarinic acid methyl Ester	C_19_H_18_O_8_	Phenolic acids	*Salvia miltiorrhiza* Bge., *Salvia przewalskii* Maxim.	Zhao C. et al. (2024)
8	Lithospermic acid B dimethyl ester	C_38_H_34_O_16_	Phenolic acids	*Salvia miltiorrhiza* Bge., *Salvia przewalskii* Maxim.	Zhao C. et al. (2024)
9	Salvianolic acid A	C_26_H_22_O_10_	Phenolic acids	*Salvia miltiorrhiza* Bge., *Salvia przewalskii* Maxim.	Zhao C. et al. (2024)
10	Danshensu methyl Ester	C_10_H_12_O_5_	Phenolic acids	*Salvia miltiorrhiza* Bge., *Salvia przewalskii* Maxim.	Zhao C. et al. (2024)
11	Salvianolic acid K	C_27_H_24_O_13_	Phenolic acids	*Salvia miltiorrhiza* Bge., *Salvia przewalskii* Maxim.	Zhao C. et al. (2024)
12	Salvianolic acid C	C_26_H_20_O_10_	Phenolic acids	*Salvia miltiorrhiza* Bge., *Salvia przewalskii* Maxim.	Zhao C. et al. (2024)
13	Caffeic acid ethyl ester	C_11_H_12_O_4_	Phenolic acids	*Salvia miltiorrhiza* Bge., *Salvia przewalskii* Maxim.	Zhao C. et al. (2024)
14	Salvianolic acid A	C_26_H_22_O_10_	Phenolic acids	*Salvia miltiorrhiza* Bge., *Salvia przewalskii* Maxim.	Zhao C. et al. (2024)
15	Chlorogenic acid	C_16_H_18_O_9_	Phenolic acids	*Salvia miltiorrhiza* Bge., *Salvia plebeia* R. Br., *Salvia chinensis* Benth.	Liang et al. (2020), Zhao C. et al. (2024)
16	Protocatechuic acid	C_7_H_6_O_4_	Phenolic acids	*Salvia miltiorrhiza* Bge.,*Salvia przewalskii* Maxim., *Salvia cavaleriei* Lévl., *Salvia cavaleriei* Lévl.	Wang (2009)
17	Tanshinone IIA	C_19_H_18_O_3_	Diterpenoid quinones	*Salvia miltiorrhiza* Bge.	Zhao C. et al. (2024)
18	Tanshinone IIB	C_19_H_18_O_4_	Diterpenoid quinones	*Salvia miltiorrhiza* Bge.	Zhao C. et al. (2024)
19	Tanshinone I	C_18_H_12_O_3_	Diterpenoid quinones	*Salvia miltiorrhiza* Bge., *Salvia glutinosa* L.	Zhao C. et al. (2024)
20	Carnosic acid	C_20_H_28_O_4_	Diterpenoids	*Salvia glutinosa* L., *Salvia miltiorrhiza* Bge.	Zhao C. et al. (2024)
21	Danshenxinkun A	C_18_H_16_O_4_	Diterpenoid quinones	*Salvia miltiorrhiza* Bge.	Bi (2023)
22	Danshenxinkun B	C_18_H_16_O_3_	Diterpenoid quinones	*Salvia miltiorrhiza* Bge.	Bi (2023)
23	Danshenxinkun C	C_16_H_12_O_3_	Diterpenoid quinones	*Salvia miltiorrhiza* Bge.	Bi (2023)
24	Danshenxinkun D	C_21_H_20_O_4_	Diterpenoid quinones	*Salvia miltiorrhiza* Bge.	Bi (2023)
25	Tanshindiol A	C_18_H_16_O_5_	Diterpenoid quinones	*Salvia miltiorrhiza* Bge.	Bi (2023)
26	Tanshinlactone	C_17_H_12_O_3_	Diterpenoid quinones	*Salvia miltiorrhiza* Bge.	Bi (2023)
27	1,2 didehydromiltirone	C_19_H_20_O_2_	Diterpenoid quinones	*Salvia miltiorrhiza* Bge.	Bi (2023)
28	Przewaquinone A	C_19_H_18_O_4_	Diterpenoid quinones	*Salvia przewalskii* Maxim.	Wang et al. (2015)
29	Cryptotanshinone	C_19_H_20_O_3_	Diterpenoid quinones	*Salvia miltiorrhiza* Bge.,*Salvia przewalskii* Maxim., *Salvia cavaleriei* Lévl.	Xun et al. (2022)
30	Miltipolone	C_19_H_24_O_3_	Diterpenoid quinones	*Salvia miltiorrhiza* Bge.	Bi (2023)
31	Danshenol B	C_22_H_26_O_4_	Diterpenoid quinones	*Salvia cavaleriei* Lévl.	Zhao et al. (2023)
32	6,7-dehydroroyleanone	C_20_H_26_O_3_	Diterpenoid quinones	*Salvia cavaleriei* Lévl.	Wang (2009)
33	Danshenol A	C_21_H_20_O_4_	Diterpenoid quinones	*Salvia cavaleriei* Lévl.	Wang (2009)
34	Ursolic acid	C_30_H_48_O_3_	Triterpenoids	*Salvia plebeia* R. Br.	Zhao C. et al. (2024)
35	Kaempferol	C_15_H_10_O_6_	Flavonoids	*Salvia plebeia* R. Br., *Salvia chinensis* Benth., *Salvia glutinosa* L., *Salvia przewalskii* Maxim., *S.* splendens Ker.-Gawl.	Zhao C. et al. (2024)
36	Quercetin	C_15_H_10_O_7_	Flavonoids	*Salvia plebeia* R. Br., *Salvia chinensis* Benth., *Salvia glutinosa* L.	Zhao C. et al. (2024)
37	Luteolin	C_15_H_10_O_6_	Flavonoids	*Salvia plebeia* R. Br., *Salvia chinensis* Benth., *Salvia cavaleriei* Lévl.	Zhao et al., 2023; Yang, 2021
38	Apigenin	C_15_H_10_O_5_	Flavonoids	*Salvia plebeia* R. Br., *Salvia glutinosa* L.	Zhao C. et al. (2024)
39	Luteolin-7-glucoside	C_21_H_20_O_11_	Flavonoid Glycosides	Salvia chinensis Benth., Salvia plebeia R. Br.	Zhao C. et al. (2024)
40	α-Pinene	C_10_H_16_	Volatile oils	*Salvia cavaleriei* Lévl., *Salvia miltiorrhiza* Bge.	Xun et al. (2022)
41	Borneol	C_10_H_18_O	Volatile oils	*Salvia cavaleriei* Lévl., *Salvia flava* Forrest ex Diels.	Zhao C. et al. (2024)
42	Camphor	C_10_H_16_O	Volatile oils	*Salvia chinensis* Benth., *Salvia deserta* Schang.	Zhao et al. (2023)
43	1-Isopropyl-4,7-dimethyl-1,2,4a,5,8,8a-hexahydronaphthalene	C_15_H_24_	Volatile oils	*Salvia cavaleriei* Lévl.	Wang (2009)
44	β-phellandrene	C_10_H_16_	Volatile oils	*Salvia przewalskii* Maxim.	Zhao et al. (2023)
45	Caryophyllene	C_15_H_24_	Volatile oils	*Salvia przewalskii* Maxim.	Zhao et al. (2023)

Selected compounds are based on their frequent occurrence across multiple species, distinctive biosynthetic origins, and/or established pharmacological significance.

**FIGURE 6:**
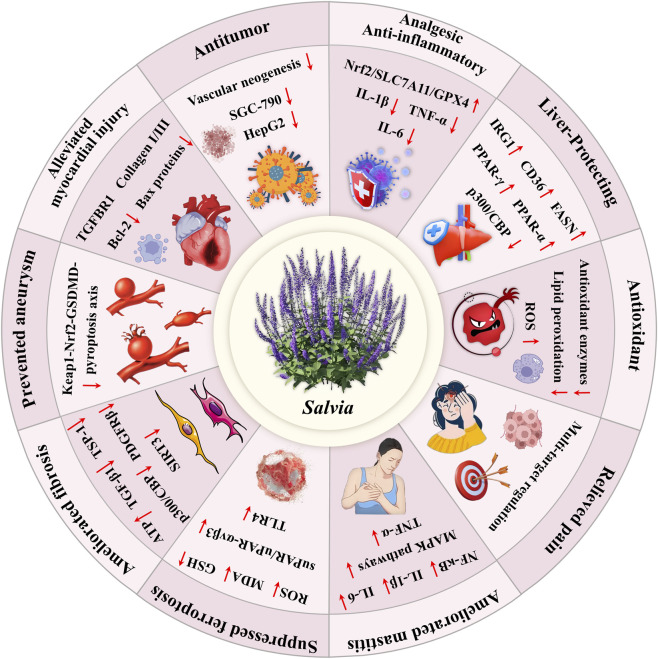
Modern pharmacological research diagram of Chinese *Salvia* plants.

Compounds included in both Figure 6 and Table 4 were selected based on the following criteria:

- Documented in at least two independent peer-reviewed studies—either within a single species or across multiple *Salvia* taxa;
- Demonstrated significant biological activity in validated *in vitro* or *in vivo* models; and/or
- Formally recognized as chemotaxonomic markers or official active ingredients in national pharmacopoeias (e.g., tanshinone IIA and salvianolic acid B in *Salvia miltiorrhiza*, as specified in the *Chinese Pharmacopoeia*, 2025 edition).

### 4.2 Functional correlation

Medicinal *Salvia* species exhibit a compelling functional linkage among their phytochemical profiles, pharmacological activities, and ecological habitats (Zhao et al., 2023; Bi, 2023; Wang, 2009). Notably, high-altitude species such as *Salvia przewalskii* are characterized by elevated levels of phenolic acids-including salvianic acid and rosmarinic acid (Li J. et al., 2020; Wang X. et al., 2019; Zhumaliyeva et al., 2023). This pattern may be attributed to environmental stressors prevalent at high elevations, particularly intense ultraviolet (UV) radiation. Supporting this hypothesis, studies on *Perilla frutescens* have demonstrated that exposure to high-intensity light significantly enhances rosmarinic acid accumulation in leaves (Xie et al., 2024), suggesting that UV-induced oxidative stress drives the biosynthesis of protective phenolic compounds in alpine plants (Chen, 2017; Yang, 2021; Lei et al., 2010; Hua, 2016).

Pharmacologically, salvianolic acids-especially salvianolic acid B-exhibit multifaceted bioactivities, including potent antioxidant, anti-inflammatory, and microcirculation-enhancing effects (Jassbi et al., 2016; Nicolescu et al., 2022). These properties directly align with traditional Tibetan medicinal applications of high-altitude *Salvia* species, which are commonly used to treat liver and gallbladder disorders-conditions frequently linked to oxidative stress and inflammation-and to promote wound healing, a process requiring coordinated regulation of inflammation, redox balance, and tissue regeneration. Indeed, experimental evidence confirms that salvianolic acid B mitigates hepatic injury and accelerates cutaneous repair through precisely these mechanisms (Lan et al., 2025; Chen et al., 2020; Bian, 2023; Chen et al., 2021).

In contrast, mid- and low-altitude species such as *Salvia chinensis* predominantly accumulate flavonoids, which underpin their use in Miao and Tujia ethnomedicine for managing gynecological inflammations and respiratory tract infections. The anti-inflammatory and antimicrobial activities of flavonoids provide a plausible phytochemical basis for these therapeutic applications (Chen et al., 2022; Wu, 2012; Xue et al., 2022; Geng et al., 2015).

Collectively, the altitudinal stratification of chemical constituents in *Salvia* reflects two interrelated principles: the “division of labor” among medicinal plant parts-where roots are typically enriched in diterpenoid quinones and phenolic acids, while aerial parts accumulate flavonoids and triterpenoids-and adaptive evolution along elevation gradients (Hu et al., 2016; Xu L. et al., 2025; Liang et al., 2020; Lou, 2023). Our analysis of 32 ethnomedicinally used *Salvia* species reveals a consistent altitudinal zonation: high-elevation taxa such as *Salvia przewalskii* accumulate UV-protective phenolic acids and are traditionally employed by Tibetan communities for “heat-clearing” and wound-healing, whereas low-elevation taxa such as *Salvia chinensis* are flavonoid-dominant and utilized by Miao and Tujia groups for inflammatory and infectious conditions. These ethnobotanical patterns, together with the documented correspondence between habitat, plant part, and chemical class, strongly support a tripartite HPPE relationship. Thus, the integration of ecological context, phytochemistry, and traditional knowledge underscores a coherent and evolutionarily informed framework for understanding the medicinal utility of Chinese *Salvia* species (Ho and Hong, 2011; Yang et al., 2020).

## 5 Pharmacological activities of active components in the *Salvia* species

The pharmacological profile of *Salvia* species is defined by pleiotropic effects driven by a diverse array of bioactive constituents-consistent with the multi-target therapeutic paradigm central to traditional botanical drugal medicine (Chen H. et al., 2024; Lopresti, 2017). Contemporary studies have reported a wide range of biological activities, including analgesic, anti-inflammatory, hepatoprotective, antioxidant, anticancer, hypoglycemic, and cardioprotective effects, among others (Liang et al., 2020; Huang J. et al., 2024).

While this breadth of activity underscores the therapeutic potential of *Salvia*, a rigorous and critical evaluation of the underlying evidence is essential to assess its methodological robustness, reproducibility, and translational feasibility. In the following subsections, we provide a focused analysis of key pharmacological actions, integrating mechanistic insights and experimental findings from *in vitro*, *in vivo*, and limited clinical studie*s.* Particular attention is given to study design limitations-such as inadequate dosing justification, lack of pharmacokinetic data, or insufficient model validation-and we conclude each section with clearly defined priorities for future research. An overview of the core pharmacological effects attributed to Chinese medicinal *Salvia* species is presented in Figure 6 and Table 5.

**TABLE 5:** Pharmacological studies of Salvia species.

Disease	Animal/cell	Modeling method	Ingredients/extract	Dosage	Outcome	Molecular mechanism	References
Anticancer activity	HepG2 cells	Cell culture	*Salvia miltiorrhiza* extract	150 μg/mL	Inhibited proliferation	Regulating cell cycle progression	Jiang Y. et al. (2017)
​	SGC-7901 cells	Cell culture	Salvianolic acid B	0.017 mmol/L	Inhibited proliferation	-	Chen et al. (2020)
​	A2780 cells	Cell culture	Cryptotanshinone	10 µM	Induced apoptosis	Activating caspase cascades	Jiang Y. et al. (2017)
​	A549 cells	Cell culture	Cryptotanshinone	-	Inhibited proliferation	Inhibiting lncRNA HOTAIR/p-Akt signaling	Zhang et al. (2021)
​	Bladder cancer cells	Cell culture	Cryptotanshinone	-	Promoted apoptosis & inhibited proliferation	Regulating PTEN/PI3K/AKT pathway	Liu H. et al. (2020)
​	Melanoma cells	Cell culture	*Salvia miltiorrhiza* extract	60 μg/mL	Suppressed growth & induced autophagy	Inhibiting STAT3 pathway	Jiang et al. (2025)
Analgesic and anti-inflammatory activity	Male Sprague-Dawley rats (24-month-old, weighing 600–700 g)	Surgical modeling	Tanshinone IIA	10 mg/kg, 20 mg/kg, or 40 mg/kg	Inhibited hippocampal inflammation & ferroptosis	Activating Nrf2/SLC7A11/GPX4 axis, tanshinone IIA treatment significantly decreased the mRNA level of prostaglandin-nndoperoxide synthase 2 (PTGS2) and increased the mRNA levels of ferritin heavy chain (FTH) and ferritin light chain (FTL) in the hippocampus of aged POCD rats	Yang et al. (2025b)
​	C57BL/6J mice	LPS induction	Tanshinone IIA	30 mg/kg	Attenuated liver injury	SIRT1/Sestrin2/HO-1 pathway, IL-1β↓, TNF-α↓	Tian et al. (2025)
​	Female Sprague-Dawley (SD) rats	The SCI group underwent SCI induction followed by saline injection	Tanshinone IIA	10 mg/kg, 20 mg/kg, and 30 mg/kg	Inhibited neuronal ferroptosis	Modulating GPX4/ACSL4 axis, ROS, MDA, and iron levels↓, GSH and SOD↑	Xu L. et al. (2025)
​	C57BL/6 mice	Immune induction	Dihydrotanshinone I	20 or 40 mg/kg	Alleviated inflammation	Directly targeting IRF3, IFN-β↓, ISGs↓, Ifnb,IL-6,TNF-α,cxcl10,Isg15 and Ifit1↓	Shi et al. (2025)
Liver-protecting activity	C57BL/6 WT mice	High-fat diet	Dihydrotanshinone I	20 mg/kg	Improved hepatic steatosis	Upregulating IRG1, CD36,FASN,SCD1,ACC,PPAR-γ,PPAR-α and CPT1a↓, ALT and AST↓	Xiang et al. (2025)
​	Mice	CCl_4_ induction	Tanshinone IIA	-	Alleviated fibrosis	Activating Nrf2/HO-1 pathway	Li Q. et al. (2024)
Antioxidant activity	Sprague–Dawley rats (male, 240–270 g)	Middle cerebral artery occlusion	15,16-Dihydrotanshinone I	7.5, 15, and 30 mg.kg−1	Inhibited ferroptosis	Activating Nrf2 pathway, ROS↓, Gpx4↓, GSH/GSSG↑	Wu et al. (2023)
​	Male SD rats	STZ induction	Salvianolic acid B	200 mg/kg, 100 mg/kg, 50 mg/kg	Reduced oxidative stress	Regulating SIRT3/FOXO1 pathway, IL-1β,IL-6,MCP-1 and TNF-α↓	Wen et al. (2023)
Other activities	Male ICR mice	The mice were anesthetized with 50 mg/kg sodium pentobarbital (i.p.) and maintained on small-animal ventilators after being secured by tape in a supine position	Tanshinone I	1, 3, and 5 mg/kg/day	Alleviated myocardial injury	Targeting TGFBR1; modulating TGF-β signaling, the aberrant expressions of collagen I/III, α-smooth muscle actin, Bcl-2, and Bax proteins↓,	Ke et al. (2025)
​	Mice	Elastase induction	Cryptotanshinone	-	Prevented aneurysm formation	Inhibiting Keap1-Nrf2-GSDMD-pyroptosis axis	Wang F. et al. (2024)
​	8-week-old pregnant Wistar rats	Bacterial infection	*Salvia miltiorrhiza* polysaccharides	87.5, 175, 350 mg/kg	Ameliorated mastitis	Suppressing NF-κB and MAPK pathways, IL-1β,IL-6 and TNF-α ↓	Zhang et al. (2022)
​	Mice	DOX (3 mg/kg body weight) was intraperitoneally administered every 3 d for a total of 7 injections (cumulative dose of 21 mg/kg) to induce nephrotoxicity	Tanshinone IIA	5 or 10 mg/kg/d	Ameliorated fibrosis	Activating SIRT3; suppressing TGF-β/TSP-1 pathway, ATP↑, TGF-β1↓, TSP-1↓, SIRT3↑	Fan et al. (2024)
​	Male C57BL/6J	STZ induction	Tanshinone IIA	10 mg/kg	Suppressed ferroptosis, protected podocytes	Modulating ELAVL1/ACSL4 signaling, ROS↓, MDA↓, GSH↑	Zhu et al. (2024)
​	Patients	Clinical PID	*Salvia*-containing formula	Clinical dose	Relieved pain	Multi-target regulation (data mining)	Liu et al. (2019)

### 5.1 Antitumor activity

*Salvia* species-particularly *Salvia miltiorrhiza*, *Salvia chinensis*, and *Salvia yunnanensis*-exhibit antitumor potential primarily through terpenoids (e.g., tanshinones), phenolic acids (e.g., salvianolic acids), and polysaccharides (Fu et al., 2017; Duan et al., 2019b; Fu et al., 2020; Zhao et al., 2015a). *In vitro*, these compounds display multimodal mechanisms: tanshinone IIA induces cell cycle arrest in HepG2 and SGC-7901 cells at low micromolar concentrations (IC_50_), triggers mitochondrial-mediated apoptosis in A2780 and A549 cells, and suppresses angiogenesis via inhibition of the HIF-1α/VEGF axis (Chen et al., 2020; Jiang Y. et al., 2017; Jiang G. et al., 2017; Wei et al., 2023; Huang W. L. et al., 2019; Zhang C. et al., 2024). Cryptotanshinone inhibits tumor growth in xenograft models by modulating the PTEN/PI3K/AKT and STAT3 pathways (Liu Y. et al., 2020; Jiang et al., 2025). The polysaccharide SMP-W1 demonstrates immunomodulatory antitumor effects in rats and exhibits antiproliferative activity against H22 hepatoma cells, albeit at a relatively high concentration of 400 μg/mL (Wei et al., 2023). Additionally, tanshinone I (0–9.6 μg/mL over 24 h) induces apoptosis and autophagy in ovarian cancer cells by inhibiting the PI3K/AKT/mTOR signaling cascade and suppresses tumor growth *in vivo* (Jin et al., 2025).

However, critical translational gaps persist. Although tanshinone IIA is used clinically in China for cardiovascular and inflammatory conditions, it is routinely tested *in vitro* at concentrations of 5–20 μM (Qin et al., 2015; Tao et al., 2025; Chen et al., 2025)-levels far exceeding those achievable in human plasma (<1 μM) due to its high lipophilicity, rapid metabolism, and poor oral bioavailability (Jing et al., 2016; Wang et al., 2020; Dai et al., 2025). Similarly, the *in vitro* efficacy of SMP-W1 at 400 μg/mL lacks pharmacokinetic validation, casting doubt on its systemic relevance.

Animal dosing regimens are often inadequately justified. For instance, one study administered a crude *Salvia miltiorrhiza* extract at 1.33 g/mL via oral gavage-a dose with no clear human equivalent-to demonstrate ovarian protection during cyclophosphamide therapy without compromising antitumor efficacy (Bi et al., 2025; Yang et al., 2025a; Yang et al., 2025b). While biologically plausible, the absence of quantified active constituents (e.g., tanshinones or salvianolic acids) limits mechanistic interpretation. Furthermore, cryptotanshinone exhibits nonlinear intestinal absorption and low baseline permeability at 100 μg/mL; its uptake is significantly enhanced only when co-administered with other tanshinones or the traditional Chinese medicine formula Danxingfang, likely via mechanisms such as P-glycoprotein inhibition (Li et al., 2015). This highlights a key limitation: studies of isolated compounds may misrepresent the true bioactivity of *Salvia*, which is traditionally used as a multi-component formulation.

Consequently, despite promising *in vitro* cytotoxicity, the majority of antitumor studies on *Salvia* Ingredientss suffer from low translational fidelity. The reliance on immunocompromised xenograft models further neglects the immunomodulatory dimension central to its traditional ethnomedicinal use.

### 5.2 Analgesic and anti-inflammatory activity

Diterpenoids (e.g., tanshinone IIA, dihydrotanshinone I) and phenolic acids (salvianolic acids A, B, C) mediate anti-inflammatory and analgesic effects primarily through suppression of pro-inflammatory cytokines (IL-6, TNF-α), attenuation of oxidative stress, and modulation of key signaling nodes-including NF-κB, the NLRP3 inflammasome, and the Nrf2 pathway (Liu H. et al., 2020; Li M. et al., 2020; Wang J. et al., 2024).

In rodent models, tanshinone IIA (10–50 mg/kg) shows therapeutic efficacy across diverse inflammatory contexts: it mitigates postoperative cognitive dysfunction via Nrf2/SLC7A11/GPX4 signaling, protects against sepsis-induced liver injury through SIRT1/Sestrin2/HO-1 activation, and reduces neuroinflammation after spinal cord trauma (Yang K. et al., 2025; Tian et al., 2025; Xu Q. et al., 2025; Li Y. et al., 2022). Dihydrotanshinone I directly inhibits IRF3, a master regulator of type I interferon responses (Shi et al., 2025).

Nevertheless, the field predominantly relies on acute systemic inflammation models-such as LPS challenge-which poorly recapitulate chronic, tissue-specific pathologies like rheumatoid arthritis or inflammatory bowel disease (Meng X. et al., 2022). Dosing regimens (10–50 mg/kg) are rarely justified by allometric scaling, and plasma or tissue exposure data are almost universally absent. Crucially, the lack of head-to-head comparisons with standard-of-care therapies (e.g., dexamethasone, NSAIDs) precludes meaningful efficacy benchmarking. Future work should prioritize target validation using genetic approaches (e.g., knockout models), adopt disease-relevant chronic models, and incorporate detailed pharmacokinetic profiling-especially for neuroinflammatory applications.

### 5.3 Liver-protecting activity

Hepatoprotection by *Salvia* species arises from the synergistic actions of diterpenoids (tanshinone IIA, cryptotanshinone), phenolic acids (salvianolic acid B, lithospermic acid), and polysaccharides (Zhang et al., 2017; Luo et al., 2024; Yin et al., 2023; Xiong et al., 2019). Key mechanisms include attenuation of oxidative stress, inhibition of the NLRP3 inflammasome, modulation of lipid metabolism, and enhancement of cellular resilience via the AMPK–SIRT1 and Nrf2 pathways (Yang et al., 2025a).

Preclinical evidence shows that pretreatment with tanshinone IIA or salvianolic acid B significantly reduces liver injury induced by CCl_4_ or acetaminophen, as evidenced by lowered serum transaminases and improved histopathology (Li Q. et al., 2024; Meng L. C. et al., 2022; Zhang G. et al., 2024; Zhang et al., 2023). In metabolic dysfunction models, these compounds ameliorate non-alcoholic fatty liver disease (NAFLD) by regulating *de novo* lipogenesis and fatty acid β-oxidation (Li S. et al., 2020; Xiang et al., 2025).

However, current studies are overwhelmingly preventive rather than interventional, with compounds administered before toxin exposure-a design that does not reflect clinical reality, where treatment typically begins after injury onset. Acute, high-dose toxin models also fail to capture the multifactorial nature of human chronic liver diseases such as NASH, which involve metabolic dysregulation, gut dysbiosis, and persistent low-grade inflammation. Moreover, testing isolated compounds ignores the synergistic matrix of whole extracts used in traditional formulas (e.g., *Danshen–Yinchenhao* decoction). Notably, none of the cited NAFLD studies assessed gut–liver axis markers (e.g., LPS, bile acids), despite emerging evidence that *Salvia* polysaccharides modulate the microbiome (Song et al., 2023; Li P. et al., 2025). Of the eight hepatoprotection studies reviewed, only two reported voucher specimens or DNA barcoding, limiting reproducibility. Thus, causal claims linking habitat-driven phytochemistry to liver efficacy remain hypothetical in the absence of controlled cultivation trials.

### 5.4 Antioxidant activity

The antioxidant capacity of *Salvia* operates through dual mechanisms: direct free radical scavenging by phenolic acids and indirect upregulation of endogenous defenses via the Keap1/Nrf2/ARE pathway. Diterpenoids (tanshinone IIA, cryptotanshinone) and salvianolic acid B mitigate oxidative damage in models of CCl_4_-induced hepatic fibrosis, cerebral ischemia-reperfusion injury, and diabetic nephropathy (Zheng et al., 2023).

Nonetheless, the physiological relevance of findings from simplistic chemical assays (e.g., DPPH, ABTS, FRAP)-which measure radical scavenging in cell-free systems-is questionable, as these conditions bear little resemblance to *in vivo* redox biology (Ma X et al., 2024; Deng et al., 2024; Meng et al., 2024). *In vivo* studies often report only one or two redox markers (e.g., MDA, GSH, SOD), providing an incomplete picture of redox homeostasis. Critically, primary antioxidant effects are rarely disentangled from secondary anti-inflammatory or metabolic outcomes; for example, Nrf2 activation may reduce oxidative stress indirectly by suppressing inflammation rather than directly quenching ROS. Furthermore, salvianolic acid B’s potent *in vitro* activity (DPPH IC_50_ ≈ 1–5 μM) has not been correlated with tissue concentrations in animal models (Ren et al., 2024; Li J. et al., 2020).

### 5.5 Other activities

Beyond core pharmacological domains, *Salvia* constituents exhibit emerging therapeutic potentials. Salvianolic acid A exerts hypoglycemic effects via AMPK activation (Qiu et al., 2024; Huang et al., 2012; Zhao C. et al., 2024), while cryptotanshinone confers cardiovascular protection by inhibiting pyroptosis in vascular smooth muscle cells (Wang T. et al., 2024; Wang et al., 2025). Certain compounds, including cryptotanshinone, display anti-virulence antimicrobial properties without inhibiting microbial growth (Chen et al., 2021). Anti-fibrotic effects in hepatic and renal tissues involve suppression of the TGF-β/Smad signaling pathway (Fan et al., 2024; Li L. et al., 2025).

However, these activities are based on isolated, preliminary findings with limited replication. For instance, cryptotanshinone’s anti-pyroptotic effect was demonstrated in a single *in vitro* model without *in vivo* confirmation or dose-response characterization. Similarly, anti-fibrotic claims rely on rodent models that do not fully recapitulate human fibrogenesis. Crucially, none of these “other activity” studies addressed habitat variation or performed comprehensive chemical profiling, thereby breaking the HPPE nexus central to this review. All lacked botanical vouchers, and dosing was often arbitrary (e.g., “10 mg/kg” without pharmacokinetic justification).

### 5.6 Verification of HPPE correlation

**FIGURE 7:**
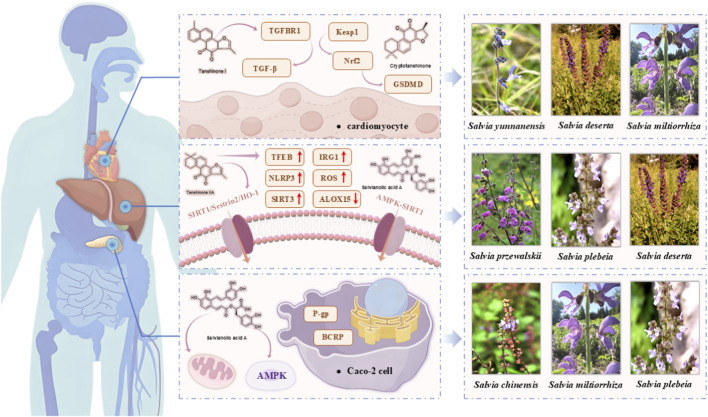
Underlying mechanisms and representative species of medicinal *Salvia*.

Emerging ecophysiological evidence suggests that environmental stressors shape phytochemical profiles and subsequent bioactivity in *Salvia* species. For example, high-altitude *Salvia przewalskii* accumulates rosmarinic acid and other phenolics, correlating with enhanced antioxidant and hepatoprotective properties consistent with its traditional medicinal uses (Fu et al., 2017). Figure 7 illustrates representative *Salvia* species and their pharmacological mechanisms.

While these observational correlations are compelling, they remain confounded by genetic variability: wild-collected samples from different regions inevitably differ in genotype, making it impossible to isolate the effects of environment alone. Robust causal validation of the HPPE model requires controlled experiments-such as common-garden or reciprocal transplant studies-using genetically uniform (e.g., clonally propagated) material across environmental gradients. To date, no such study has been conducted in *Salvia*. Furthermore, bioactivity assessments in correlative studies have relied on non-standardized extracts, preventing direct linkage between measured Ingredientss (e.g., rosmarinic acid) and observed effects. Only one study integrated untargeted metabolomics with standardized bioassays. Until controlled ecophysiological trials are implemented, the HPPE framework-though conceptually powerful-lacks causal empirical support in *Salvia*.

## 6 Pharmacological activities of classic compound formulations

Currently, clinical applications based on *Salvia* species primarily focus on formulations included in pharmacopoeias or widely adopted in conventional practice (e.g., Danshen Injection and Compound Danshen Dripping Pills). However, numerous ethnic medicinal preparations-such as *Salvia przewalskii* ointment and Yi medicine *Salvia yunnanensis* external patches-remain under-researched. Despite their historical use in folk medicine, these formulations lack rigorous clinical validation through modern evidence-based standards. Systematic evaluation of their efficacy, safety, and pharmacokinetic profiles is critically needed to bridge traditional practice with contemporary regulatory requirements.

### 6.1 Cardiovascular diseases

*Salvia*-containing formulations represent cornerstone therapies for cardiovascular disorders. Key preparations include Danshen Injection, Danhong Injection, Compound Danshen Dripping Pills, and Shuang Shen Ning Xin Formula. These exhibit therapeutic effects through bioactive constituents like tanshinones and salvianolic acids, modulating inflammation, oxidative stress, autophagy, and critical signaling pathways (e.g., NF-κB, TXNIP/NLRP3/Caspase-1, AMPK, STING-TBK1) (Zhang X. et al., 2025).

As a classical monotherapy, Danshen Injection targets coronary heart disease with Qi stagnation and blood stasis syndrome via metabolic pathway regulation (Wu et al., 2025). Danhong Injection, a synergistic combination of *Salvia miltiorrhiza* and *Carthamus tinctorius* (Honghua), demonstrates neuroprotective effects in ischemic stroke by modulating microglial polarization and neuroinflammation through the JUNB/NF-κB axis. Notably, its combination with hyperbaric oxygen therapy significantly improves 90-day functional outcomes and reduces 1-year recurrence rates in ischemic stroke patients (Xie et al., 2025; Zheng and Xu, 2025).

Compound Danshen Dripping Pills exhibit robust efficacy in stable angina pectoris. Mechanistically, this formulation attenuates myocardial infarction through metabolic trajectory modulation, inhibits vascular calcification via multi-pathway suppression, and disrupts STING-TBK1 interactions to mitigate cGAS-STING-driven pathology (Liu and Zhu, 2025; Zhang C. J. et al., 2024; Yang et al., 2024; Shi et al., 2024; Zhao et al., 2015b). Additional evidence highlights Salvia-based decoctions’ cardioprotective roles: A Danshen decoction targets miR-93-5p to regulate the TXNIP/NLRP3/Caspase-1 pathway in myocardial ischemia-reperfusion injury (Chen M. et al., 2024), while Huangqi-Danshen (Astragalus-Salvia) decoction ameliorates heart failure via pericardial adipose-derived extracellular vesicle miR-27a-3p-mediated AMPKα2 mitophagy activation (Chen Z. et al., 2024). Shuang Shen Ning Xin Formula’s anti-myocardial ischemia-reperfusion injury (MI/RI) effects are corroborated by mass spectrometry imaging (Chen H. et al., 2024; Ezema et al., 2022; Li L. et al., 2024), and Guanxinning Injection attenuates cardiac fibrosis in heart failure models through SLC7A11/GPX4 axis modulation (Wang C. et al., 2023).

In summary, *Salvia*-based formulations demonstrate significant multi-Ingredients, multi-target therapeutic efficacy against cardiovascular diseases-including coronary heart disease, ischemic stroke, myocardial infarction, and heart failure-through integrated regulation of inflammatory, oxidative stress, metabolic, and signaling networks. These mechanisms underscore their substantial clinical translational potential.

### 6.2 Gynecological and reproductive system diseases

In Chinese clinical practice, *Salvia*-containing gynecological formulations (e.g., Dan’e Fukang Decoction Extract) are widely utilized, with select preparations standardized in national pharmacopoeias. Key products include Compound Salvia Injection, the *Salvia-Panax notoginseng* botanical drug pair, and *Salvia-Ligustrazine* combinations. Their therapeutic mechanisms involve inflammatory modulation, hemodynamic improvement, and targeted signaling pathway regulation.

The *Salvia-Panax notoginseng* botanical drug pair alleviates gynecological blood stasis syndromes by blocking arachidonic acid-prostaglandin conversion (Zeng et al., 2024). Compound Salvia Injection demonstrates multifaceted efficacy: It regulates aquaporin 3 expression in human amniotic epithelial cells via MAPK signaling (Zhang et al., 2013; Ma et al., 2013), synergizes with magnesium sulfate to improve maternal-infant outcomes in pregnancy-induced hypertension (Zhao et al., 2021), and enhances perinatal outcomes when combined with low-molecular-weight heparin calcium in early-onset severe preeclampsia complicated by nephrotic syndrome (Tong et al., 2015).

In addition, *Salvia* Injection combined with chitosan for fallopian tube recanalization can improve the long-term patency rate of fallopian tubes and alleviate infertility symptoms (Huang C. et al., 2019); *S.* miltiorrhiza-Ligustrazine is more effective than ligustrazine alone in treating gestational hypertension (Ji et al., 2024); a comprehensive regimen containing Salvia can prevent re-adhesion after surgery for moderate-to-severe intrauterine adhesions (Pan et al., 2021). In the treatment of chronic pelvic pain caused by chronic pelvic inflammatory disease, prescriptions containing Salvia are also one of the commonly used regimens (Liu et al., 2019).

In summary, preparations containing Chinese medicinal *Salvia* species exhibit significant therapeutic effects in gynecological and reproductive system diseases, such as pelvic inflammatory disease, pregnancy-induced hypertension, pre-eclampsia, intrauterine adhesions, and infertility. Their mechanisms of action are multi-Ingredients and multi-targeted, including regulating inflammatory responses, improving blood stasis, modulating signaling pathways (e.g., MAPK), and enhancing tissue repair and patency. These preparations, some of which are included in national standards, have shown reliable efficacy in clinical practice, underscoring their important clinical value in the field of gynecology.

### 6.3 Pharmacological activities of other diseases

Beyond cardiovascular and gynecological applications, *Salvia* demonstrates diverse clinical utility in musculoskeletal, pulmonary, immunological, renal, and metabolic disorders.

#### 6.3.1 Musculoskeletal and pulmonary diseases

Tanshinone IIA-loaded liposomes mitigate acute muscle injury via autophagy enhancement and oxidative stress reduction (Wang et al., 2020). Astragalus-*Salvia* injection synergistically treats skeletal muscle injury in athletes (Wei and Jinguo, 2018). XueBiJing injection alleviates sepsis-induced acute lung injury through Hippo pathway-mediated suppression of oxidative stress, ferroptosis, apoptosis, and inflammation (Zou et al., 2025; Ling et al., 2025). Danhong Injection prevents LPS-induced acute lung injury in mice (Wan et al., 2013), while Cordyceps-Astragalus-*Salvia* combinations treat paraquat-induced lung injury in rats (Li et al., 2017). For pulmonary fibrosis, *Salvia*-Ligustrazine combinations inhibit TGF-β-driven myofibroblast differentiation and modulate TNF-α/TGF-β1 axes (Shao et al., 2019; Huang et al., 2013).

#### 6.3.2 Immunological and renal disorders

Astragalus-*Salvia* and Ophiopogon-Dendrobium botanical drug pairs alleviate Sjögren’s syndrome in NOD/Ltj mice via JAK1/STAT3 and PI3K/AKT pathway inhibition (Sun et al., 2025). Huangqi-*Salvia* decoction targets SCD1 to modulate cGAS/STING signaling and inhibit TGF-β1 pathways in renal fibrosis (Peng et al., 2025; Huang X. et al., 2024). Astragalus-*Salvia* combinations mitigate cyclosporin A-induced nephrotoxicity through lipid metabolism regulation (Gao et al., 2025) and demonstrate multi-target efficacy in diabetic nephropathy (Liang et al., 2025; Abd Rashed and Rathi, 2021).

#### 6.3.3 Neurological, oncological, and metabolic applications

**FIGURE 8:**
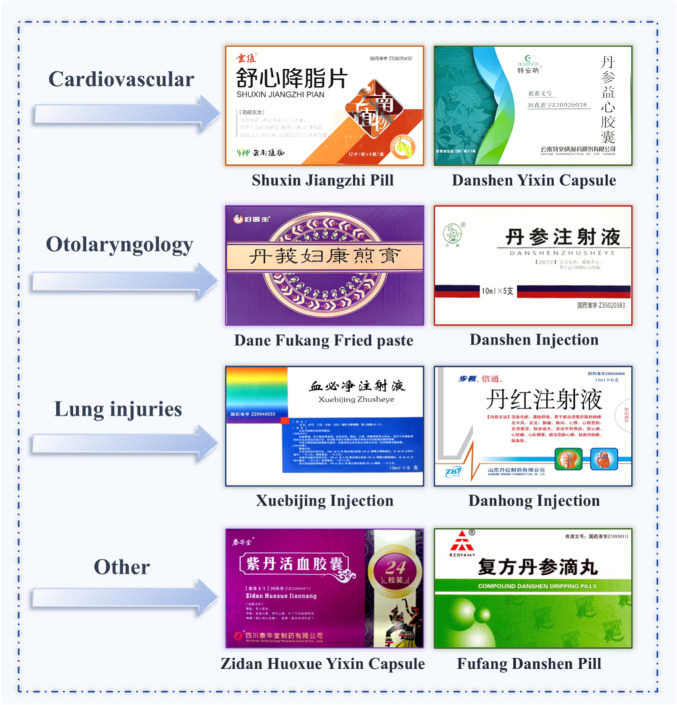
Representative classic compound diagram containing Chinese medicinal *Salvia*.

*Salvia*-Ligustrazine injection improves acute cerebral infarction outcomes (Ma Z. et al., 2024), while *Salvia* injection induces AML cell apoptosis via p38MAPK activation (Zhong et al., 2025). Compound Danshen Pills regulate metabolic profiles in hypercholesterolemia (Li S. et al., 2025), and *Salvia*-Reynoutria japonica combinations alleviate non-alcoholic fatty liver disease (Chen S. et al., 2024). Panax notoginseng-*Salvia* Ingredientss show melasma therapeutic potential (Wei et al., 2025). Figure 8 presents a representative classic compound including Chinese medicinal *Salvia*.

## 7 Results

Our integrated analysis of 32 ethnomedicinal *Salvia* species validates the hypothesis that “altitudinal adaptation drives chemical differentiation, which in turn shapes medicinal practice.” This study systematically elucidates the intrinsic linkage among ethnic application-habitat adaptation-compound accumulation-efficacy manifestation.

Ethnobotanical Patterns: *Salvia* species are predominantly distributed in Southwest China, with 17 ethnic groups exhibiting habitat-adapted medicinal practices. Tibetan communities primarily utilize high-altitude species (e.g., *Salvia przewalskii*) to treat plateau-specific disorders, whereas Miao and Tujia groups predominantly rely on low-altitude whole-plant species for damp-heat-related ailments. A clear pattern in medicinal part selection emerged: roots (approximately 60% of usage), rich in diterpenoids and phenolic acids, are primarily used for promoting blood circulation and resolving stasis; whole plants (approximately 40%), abundant in flavonoids and triterpenoids, are mainly employed for clearing heat and detoxification.

Biological and Chemical Divergence: Elevation serves as a key driver of chemical differentiation. Low-altitude species (e.g., *Salvia miltiorrhiza*) flower in spring, accumulate flavonoids, and are widely cultivated. In contrast, high-altitude species (e.g., *Salvia przewalskii*) flower in summer and accumulate phenolic acids as UV-protective antioxidants, though their cultivation remains challenging. This chemical divergence is underpinned by genetic mechanisms, including altitude-driven functional differentiation of *CYP76AK* enzymes.

Cross-species analysis supports a coherent “plant part-chemical class-pharmacological action” correspondence. Lipophilic diterpenoid quinones (abundant in roots of *Salvia miltiorrhiza* and *Salvia przewalskii*) exhibit antitumor and cardiovascular-protective activities. Hydrophilic phenolic acids (enriched in high-altitude species such as Salvia przewalskii) demonstrate potent antioxidant and anti-inflammatory effects. Flavonoids and triterpenoids from aerial parts (characteristic of whole-plant species like *Salvia plebeia* and *Salvia chinensis*) contribute to antimicrobial and immunomodulatory functions. This evidence substantiates the proposed linkage: environmental adaptation shapes chemical profiles, which in turn underpins both pharmacological activity and traditional therapeutic applications.

## 8 Discussion

Across all pharmacological domains, less than 30% of studies met high-evidence criteria per GA checklist, primarily due to inadequate botanical authentication, uncharacterized extracts, and non-physiological dosing. The majority were downgraded due to inadequate botanical authentication-such as the absence of voucher specimens or molecular identification-or the use of chemically uncharacterized crude extracts. These methodological shortcomings substantially undermine the reliability of current claims linking habitat variation to pharmacological efficacy in ethnomedicinal *Salvia* species.

In response to these gaps, this review systematically integrates multidimensional evidence-including ecological, phytochemical, and pharmacological data-to establish and validate a coherent HPPF linkage model for ethnomedicinal *Salvia* species in China. Collectively, the findings support our central hypothesis: altitudinal gradients and associated environmental stressors serve as key drivers of chemical diversification, which in turn modulates pharmacological activity and ultimately informs traditional ethnomedicinal uses. The following discussion contextualizes this synthesized model within the broader scientific literature, explores its theoretical and practical implications, acknowledges inherent limitations, and proposes concrete directions for future research.

### 8.1 The HPPE model as a multidisciplinary bridge

Our proposed model provides a robust framework that transcends disciplinary boundaries, connecting ethnobotany, ecology, phytochemistry, and pharmacology. It moves beyond the descriptive cataloging of traditional uses or isolated compound activities, offering a causative explanation for why specific *Salvia* species were empirically selected by different ethnic groups. The clear altitudinal zonation-with Tibetan medicine favoring high-altitude, phenolic acid-rich species for “heat-clearing” and wound healing, and lowland ethnic groups using flavonoid-enriched species for inflammatory conditions-exemplifies a rational, habitat-adapted pharmacopoeia. This pattern strongly aligns with the traditional medical tenet of “using locally sourced materials”, suggesting that indigenous knowledge systems intuitively recognized and exploited plant-environment-chemical interactions long before modern scientific inquiry.

### 8.2 Environmental drivers and chemodiversity: from correlation to mechanism

The correlation between altitude and chemical class accumulation (phenolic acids vs. flavonoids) is a pivotal finding. We posit that this is not merely correlative but reflects a direct adaptive response. High-altitude environments, characterized by intense UV-B radiation and temperature fluctuations, impose significant oxidative stress. The biosynthesis of phenolic acids, particularly rosmarinic and salvianolic acids, is a well-documented plant defense mechanism against such stressors, functioning as potent antioxidants and UV-absorbing compounds. Our analysis suggests that ethnic groups inhabiting these regions (e.g., Tibetans) selectively utilized these chemically “pre-adapted” plants for treating conditions involving oxidative damage and inflammation, such as liver disorders and slow-healing wounds. Conversely, the flavonoid-dominant profile of low-altitude species may be more attuned to pathogen defense in warmer, humid climates, explaining their use for infections and superficial inflammation by Miao and Tujia communities. This mechanistic link between an abiotic stressor (UV), a biochemical response (phenolic acid synthesis), and a therapeutic outcome (antioxidant/anti-inflammatory effect) provides a powerful validation of the model’s predictive power.

However, this very adaptation poses significant sustainability and cultivation challenges. High-altitude species often require specific environmental conditions-such as low temperatures, intense UV exposure, and particular soil properties-to synthesize these target compounds. This makes large-scale cultivation and a sustainable resource supply difficult, especially for species with narrow ecological niches. Future efforts toward domestication or controlled cultivation must therefore carefully replicate these abiotic stressors to maintain chemoprofile integrity, adding complexity to conservation and commercial use.

### 8.3 Functional compartmentalization and the logic of medicinal part selection

The observed chemical partitioning between roots (diterpenoid quinones, phenolic acids) and aerial parts (flavonoids, triterpenoids) offers a phytochemical rationale for the traditional practice of using different plant parts for different ailments. The roots’ focus on lipophilic diterpenes (involved in complex mammalian signaling pathways like PI3K/AKT and STAT3) and systemic antioxidants aligns with treating internal, chronic, and “stasis-related” disorders like cardiovascular disease and tumors. The aerial parts’ repertoire of antimicrobial flavonoids and immunomodulatory triterpenoids suits them for addressing external, acute, “heat-toxin” conditions like infections and trauma. This “division of labor” underscores a sophisticated understanding of plant chemistry in traditional medicine systems, which our model helps to decode in modern pharmacological terms.

### 8.4 Limitations of current evidence and future research priorities

Despite its integrative strengths, this study faces critical limitations that guide future research priorities. First, the molecular mechanisms underlying high-altitude adaptations in Salvia species (e.g., *Salvia przewalskii*) remain mechanistically opaque. While phenolic acids and diterpenoid quinones (e.g., przewaquinone A) correlate with antioxidant and anti-inflammatory activities, the precise pathways mediating hypoxia tolerance-such as HIF-1α stabilization, NLRP3 inflammasome suppression, or macrophage polarization-lack rigorous validation. Most supporting evidence derives from single-laboratory *in vitro* studies using non-standardized extracts, limiting reproducibility and biological interpretability. Systematic dissection through multi-omics integration (transcriptomics, metabolomics) coupled with genetic perturbation (e.g., CRISPR/Cas9 or siRNA knockdown) is essential to establish causal links between habitat-driven phytochemical shifts and functional outcomes.

Second, a stark disconnect persists between ethnopharmacological practices and evidence-based validation. Many ethnic-specific formulations-such as Tibetan *Salvia*-based ointments or Mongolian decoctions-remain undocumented in peer-reviewed literature or are evaluated only in small, uncontrolled clinical observations. This gap not only hinders global recognition but also raises concerns about intellectual property protection and benefit-sharing with indigenous communities. Future efforts must prioritize well-designed pharmacological and clinical studies that respect traditional knowledge while adhering to international standards (e.g., CONSORT for trials, STROBE for observational studies), ensuring both scientific rigor and ethical integrity.

Third, the therapeutic potential of key *Salvia* actives-particularly lipophilic tanshinones like tanshinone IIA-is severely constrained by poor aqueous solubility, low oral bioavailability, and rapid metabolism. Yet, few studies integrate pharmacokinetic profiling with formulation science. Bridging this gap requires interdisciplinary innovation: advanced drug delivery systems (e.g., nanoparticles, phospholipid complexes, or micelles) should be co-developed with standardized extracts traceable to pharmacopeial references (e.g., *Chinese Pharmacopoeia*), enabling reliable dose–response assessments and meaningful comparisons across studies.

Finally, while the HPPE model offers a compelling framework for understanding ecological drivers of medicinal quality, its generalizability remains untested. Expanding this paradigm to other ethnomedicinal genera (e.g., Rhodiola, Codonopsis, or Gentiana) could establish a predictive, ecology-informed strategy for sustainable bioprospecting. However, such expansion demands harmonized protocols for plant authentication, chemical standardization, and environmental metadata collection-ideally aligned with Good Agronomic and Collection Practices (GACP) and the GA Online reporting standards.

Addressing these challenges through collaborative, interdisciplinary strategies-spanning ethnobotany, analytical chemistry, systems pharmacology, and translational medicine-will be pivotal to advancing both mechanistic insight and real-world applications of plant-environment-chemistry interactions in global health.

### 8.5 Conclusion

In conclusion, this multidimensional analysis substantiates the HPPE paradigm as a critical framework for understanding and developing ethnomedicinal resources. It demonstrates that the traditional applications of Salvia are not arbitrary but are deeply rooted in the plants’ ecological adaptations and their resulting chemical profiles. By validating this linkage, our work not only provides a scientific rationale for traditional practices but also establishes a forward-looking, mechanism-driven roadmap for the conservation, quality-focused cultivation, and targeted pharmacological development of *Salvia* species-and potentially other medicinal plants-within their ecological contexts.

However, this study has limitations in elucidating the genetic mechanisms underlying the “habitat-constituent” relationship. Although we cited research on the functional differentiation of CYP76AK enzymes (Hu et al., 2023) to illustrate a genetic basis for chemical diversity, current evidence does not firmly establish a direct causal link between altitudinal gradients and the adaptive differentiation of specific enzyme genes. The genetic drivers of the observed altitudinal patterns in chemical composition are likely polygenic and multifaceted. Future studies should integrate eco-genomic approaches-such as whole-genome resequencing of populations across altitudes to detect altitude-associated selection signals, common-garden experiments combined with metabolomic and transcriptomic analyses to dissect environmental effects on gene expression and Ingredients accumulation under controlled genetic backgrounds, and gene-editing techniques for functional validation of candidate genes. Only through such integrated strategies can we advance from describing correlations to revealing causal mechanisms.
